# Effective Ion Concentration as a Descriptor for the Local Reaction Environment at Nanoparticle-Based Electrocatalysts

**DOI:** 10.1021/acscatal.5c06754

**Published:** 2026-02-10

**Authors:** Yufan Zhang, Tobias Binninger, Jun Huang, Michael Eikerling

**Affiliations:** † Theory and Computation of Energy Materials (IET-3), Institute of Energy Technologies, 28334 Forschungszentrum Jülich GmbH, Jülich 52425, Germany; ‡ Chair of Theory and Computation of Energy Materials, Faculty of Georesources and Materials Engineering, RWTH Aachen University, Aachen 52062, Germany

**Keywords:** supported electrocatalysts, local reaction environment, electro-ionic metal−support interactions, electrical double layer, Frumkin correction, proton concentration

## Abstract

Electrocatalyst nanoparticles, attached to an electronically conductive support material, are key components that determine the performance and lifetime of electrochemical devices like fuel cells and electrolyzers. Differences in electronic and electrochemical properties between nanoparticles and support induce phenomena subsumed as electro-ionic metal–support interactions. These phenomena are responsible for heterogeneously distributed electron densities and electrical double-layer properties over the surface. The resulting local reaction environment (LRE), qualitatively different from that of single-crystalline extended surfaces, remains poorly understood. In an effort to address this shortcoming, the current work introduces the effective ion concentration as a quantitative descriptor for the LRE around supported nanoparticles. This property is defined as the average ion concentration over the reaction plane. Using gold-supported silver nanoparticles immersed in acidic solutions as a model system, we investigate how the effective proton concentration depends on the size and the packing density of nanoparticles, Fermi levels of nanoparticle and support materials, bulk electrolyte concentrations, and electrode potential. To further rationalize its impact on electrocatalytic activity, we define a complementary LRE descriptor that incorporates the effect of the local electrostatic potential. Based thereon, an activity descriptor is introduced by combining the two reaction-agnostic LRE descriptors with two reaction-specific kinetic parameters, *viz.*, reaction order and transfer coefficient. Results are discussed in view of the suitability of the descriptors to be used in the design and optimization of nanoparticle-based electrocatalysts for electrochemical applications.

## Graphical abstract

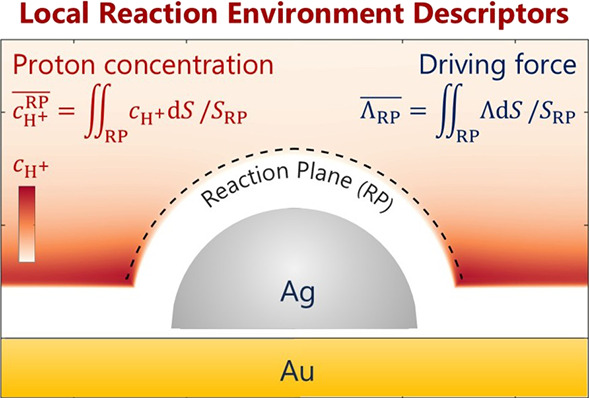

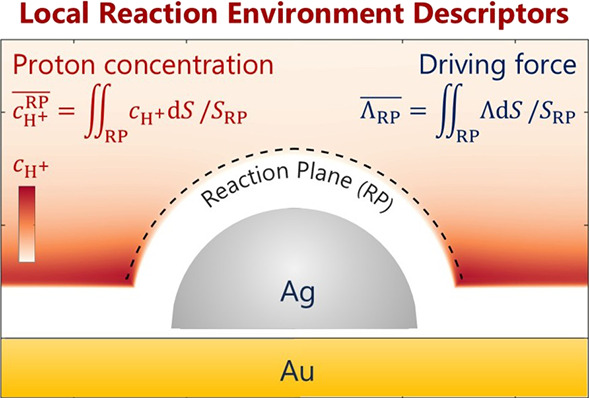

## Introduction

Driven by the ever more apparent and severe consequences of climate change and the depletion of natural resources, the global energy infrastructure is undergoing a deep transformation. Bringing about the required shift toward a defossilized and sustainable economy demands the rapid development of highly performing, cost-effective, and durable energy conversion technologies, including hydrogen fuel cells and water electrolyzers.^1–3^ Electrochemical conversion processes in these devices hinge on transition metal electrocatalysts to accelerate specific reaction pathways. Since heterogeneous catalytic reactions entail surface processes, electrocatalysts are prepared preferably as nanoparticles (NPs) that yield a high surface-area-to-volume ratio.^4^ This allows for greater utilization of a given material on a mass basis, thereby reducing overall loading and cost. NPs are supported on high surface-area porous substrates that must be electronically conductive, confer high chemical and mechanical stability, provide stable anchoring of NPs, and ideally, should also help enhance electrocatalytic activity.^5^ Supported electrocatalysts are thus central to modern chemical conversion technologies, adding complexity to materials design and device engineering. Notable examples include carbon-supported platinum NPs for the oxygen reduction reaction (ORR),^6^ oxide-supported iridium-based NPs for the oxygen evolution reaction (OER),^3^ and carbon supported coinage metals for the carbon dioxide reduction reduction (CO_2_RR).^7^

Several factors govern the catalytic activity of supported electrocatalysts. Decreasing NP size alters the proportion of exposed crystal facets.^8,9^ Smaller NPs exhibit a larger proportion of under-coordinated edge and corner sites whereas larger particles have more terrace sites.^10,11^ Since adsorption strengths of reaction intermediates vary with crystallographic orientation and surface atom coordination, catalytic activities exhibit pronounced NP size effects.^1,12^ In addition, the support material alters the catalytic performance through interfacial phenomena subsumed somewhat vaguely as metal–support interactions (MSI).^13^ These include electron redistribution,^14–17^ spillover of reaction intermediates,^18–20^ lattice strain induced by mismatch at interfacial sites,^21–23^ and partial encapsulation of the NP surface by oxide support.^24–26^ These effects are often entangled and vary in magnitude depending on the specific system and materials of interest.

In contrast to heterogeneous catalytic reactions for which the catalytic activity is primarily determined by electronic properties and adsorption energetics, electrocatalytic reactions are influenced by the local reaction environment (LRE).^27^ The LRE refers to the electric potential, electric field, and ion concentrations at the reaction plane or within the reaction region, all of which are governed by the structure of the electrical double layer (EDL).^28^ The influence of the LRE on the reaction kinetics is broadly referred to as the *Frumkin effect*, and incorporating these factors (or a subset of them) into kinetic expressions is referred to as applying the *Frumkin correction*.^28,29^ A defining feature of electrochemical systems is that the surface charge density at the electrode surface can be tuned by the electrode potential, as entailed in the charging relation. In textbook-style electrochemistry, the charging relation is determined by two parameters, the potential of zero charge (PZC) and the differential capacitance. When the electrode potential is tuned away from the PZC, the surface accumulates excess charges in proportion to the differential capacitance.

As NP and support are typically made of different materials, they often exhibit dissimilar PZC and differential capacitance. As a result, the EDL structures at the NP–solution interface and the support–solution interface differ. Due to the nanoscale dimension, these two EDLs overlap, resulting in a jointly determined heterogeneous LRE.^30,31^

The importance of the EDL overlap effect in supported NP systems has garnered attention over the past decade.^30–33^ For instance, for Pt NPs dispersed on planar or porous carbon supports, experimental results reveal a high sensitivity of the ORR activity to the interparticle distance.^30,32,33^ When the interparticle distance is reduced, while the NP size remains constant, the ORR activity per real catalyst surface area increases, a phenomenon known as the particle proximity effect. Closer proximity is equivalent to an increaseof theNP-covered support surface fraction (NCSF) on the support. Early investigations by Nesselberger et al. and Huang et al. established the EDL overlap as an underlying mechanism for the particle proximity effect, albeit with differing explanations.^30,31^

Nesselberger et al. attributed the NP proximity effect to an increased overlap of EDLs from neighboring Pt NPs at smaller interparticle distances.^30^ This overlap reduces the potential drop from electrode surface to the inner Helmholtz plane, leading to a weaker electric field in the compact layer of the EDL. A reduced electric field was assumed to lower the coverage of site-blocking oxygen-containing adsorbates, thereby facilitating the ORR. While this study experimentally demonstrated the importance of EDL overlap, the model has several limitations. Firstly, it assumed an identical PZC for Pt and carbon, overlooking their dissimilar charging characteristics. Secondly, in reality, adsorbed oxygen-containing adsorbates not only act as site-blocking species, as assumed in the study, but also participate as reactants. Therefore, a simple inverse relationship between adsorbate coverage and catalytic performance may not always hold.

To address these limitations, the model of Huang et al. not only accounts for the distinct surface charging behaviors of Pt NP and carbon support, but also explicitly describes the formation of oxygen-containing adsorbates, and rationalizes the net impact of these effects on the LRE.^31^ Within the potential range relevant to the ORR, the carbon support is positively charged, leading to proton exclusion. On the Pt surface, however, the nonmonotonic surface charging behavior that had been found in an earlier work results in a net negative charge in the relevant potential regime, which leads to proton attraction.^34^ Dissimilar charging properties of Pt NP and support jointly determine the EDL structure. Huang et al. thus attributed the particle proximity effect to changes in the local proton concentration, which are regulated by the interplay of the surface charging characteristics of Pt NPs and the support and depend on the NCSF. At higher NCSF, electrostatic properties of Pt NPs become more dominant and induce higher proton concentrations. A simple Butler–Volmer-type equation was used to calculate the ORR activity.^31^ In a subsequent study by Zhang et al., a more detailed microkinetic model that accounts for four reaction steps of the ORR was combined with the EDL overlap model to calculate reaction rates and construct electrocatalytic volcano plots.^35^ This modeling work explored how the volcano curve can be shifted upward or downward via altering the support material or the bulk electrolyte concentration. The position of the volcano apex remained, however, unchanged upon applying these modifications.

A limitation, nowadays well-recognized, of the aforementioned approaches, is their exclusive focus on the electrolyte side, whereas metal electrons are not explicitly accounted for and the metal is treated through a charging boundary condition. However, when electrocatalyst NP and support come into contact, the difference in their Fermi levels drives electron redistribution across the solid–solid interface until thermodynamic equilibrium is reached and the Fermi levels become equalized. Without explicit treatment of electrons, this electron redistribution effect, which modulates the charging response of the combined system, could not be adequately accounted for. Importantly, the redistribution of electron density in the electronic subsystem and the EDL overlap in the ionic subsystem are correlated and jointly determine the LRE, demanding electronic and ionic degrees of freedom to be considered on an equal footing and in a self-consistent manner.

To fill this gap, Huang et al. developed an advanced theoretical framework called density potential functional theory (DPFT).^36–38^ The approach combines orbital-free density functional theory (OF-DFT) for metal electrons^39–41^ with statistical field theory for electrolyte species in the EDL.^42–44^ Notably, DPFT enables constant-potential simulations by fixing the electrochemical potential of electrons. Using this framework, Zhang et al. studied a model system consisting of silver (Ag) NPs on a polycrystalline gold (pcAu) support.^36^ Results revealed that the charging behavior and LRE of supported NP electrodes are qualitatively different from that of planar extended surfaces. The PZC of the NP cannot guarantee either zero electronic charge or zero ionic charge of the NP surface due to the combined effect of electron redistribution and double layer overlap. In response, two new characteristic electrode potentials are introduced, namely the potential of zero local electronic charge (PZLeC) and the potential of zero local ionic charge (PZLiC). For the system studied, PZLeC and PZLiC can deviate from the classical PZC in opposite directions by up to 0.6 V. As the qualitative differences arise from interactions between the electronic subsystem and the ionic subsystem, the underlying mechanism is thus termed *electro-ionic metal–support interaction (EIMSI)*.

Despite recent progress, it remains poorly understood how NP size, NCSF, support material, and electrolyte composition determine the EIMSI and the LRE in supported electrocatalyst systems. Gaining a better understanding of these dependencies will be essential for the rational design of electrocatalyst materials for electrochemical devices with high performance and durability.

In this work, we address this need by evaluating the effective proton concentration as the average proton concentration over the reaction plane (RP) of supported NPs, using the solution of the DPFT model. For proton-consuming reactions that occur on the cathode side of polymer electrolyte fuel cells, *viz*., ORR and catalyst dissolution, we expect this effective proton concentration to serve as a meaningful LRE descriptor. This composite interface descriptor establishes the missing linkage between LRE effects, as obtained from microscopic theories^45,46^ and simulations,^47,48^ and device level metrics related to performance, lifetime, and economic viability.^49,50^ We will systematically evaluate how structural, compositional and operational parameters affect the newly proposed descriptor. We then propose a complementary LRE descriptor that accounts for the impact of the local electrostatic potential, and analyze how two key kinetic parameters, namely the reaction order w.r.t. the proton concentration, γ_H^+^_, and the electronic transfer coefficient, *α*, govern the relationship between LRE descriptors and catalytic activity.

The structure of the article is as follows. We first define our model system, present the theoretical framework, and introduce the LRE descriptor. This is followed by a parametric analysis exploring the key structural and compositional properties and operating conditions. Furthermore, we extend the analysis to establish how relevant LRE effects can be combined into an activity descriptor that properly reproduces and eventually allows predicting trends of reaction rates. Finally, we conclude with a summary of the main findings and their implications for the optimization of supported electrocatalysts.

## Methods

## Model System

Building on our previous work, in which DPFT was successfully applied to a model system consisting of Ag NPs on a pc Au surface,^36^ we herein further investigate how the EDL overlap effect influences the LRE. The consideration of a system of Au-supported Ag NP rather than technologically more relevant systems such as carbon-supported Pt NP is owed to methodological limitations of DPFT. Simulating Pt-based catalysts poses challenges due to chemisorption phenomena in the potential range above 0.6 V_SHE_, which lie beyond the capabilities of the current DPFT framework.^51^ On the other hand, simulating carbon supports is complicated by their low density of electronic states near the Fermi level, which hinders the applicability of the DPFT framework primarily developed for metal electrodes. In contrast, the Ag/Au system offers a tractable yet physically meaningful model that retains essential characteristics of supported electrocatalysts while remaining computationally feasible within DPFT.

As shown in Figure 1a, the model system features a hemispherical Ag NP attached to a planar Au support. The axial symmetry of the system allows representation using a radial coordinate *r* and a surface-normal coordinate *z*. The radius of the hemispherical NP, *R*_cat_, and the radial size of the simulation cell, *R*_cell_, define the NCSF as $\theta =R_{\mathrm{cat}}^{2}/R_{\mathrm{cell}}^{2}$ .

**Figure 1:**
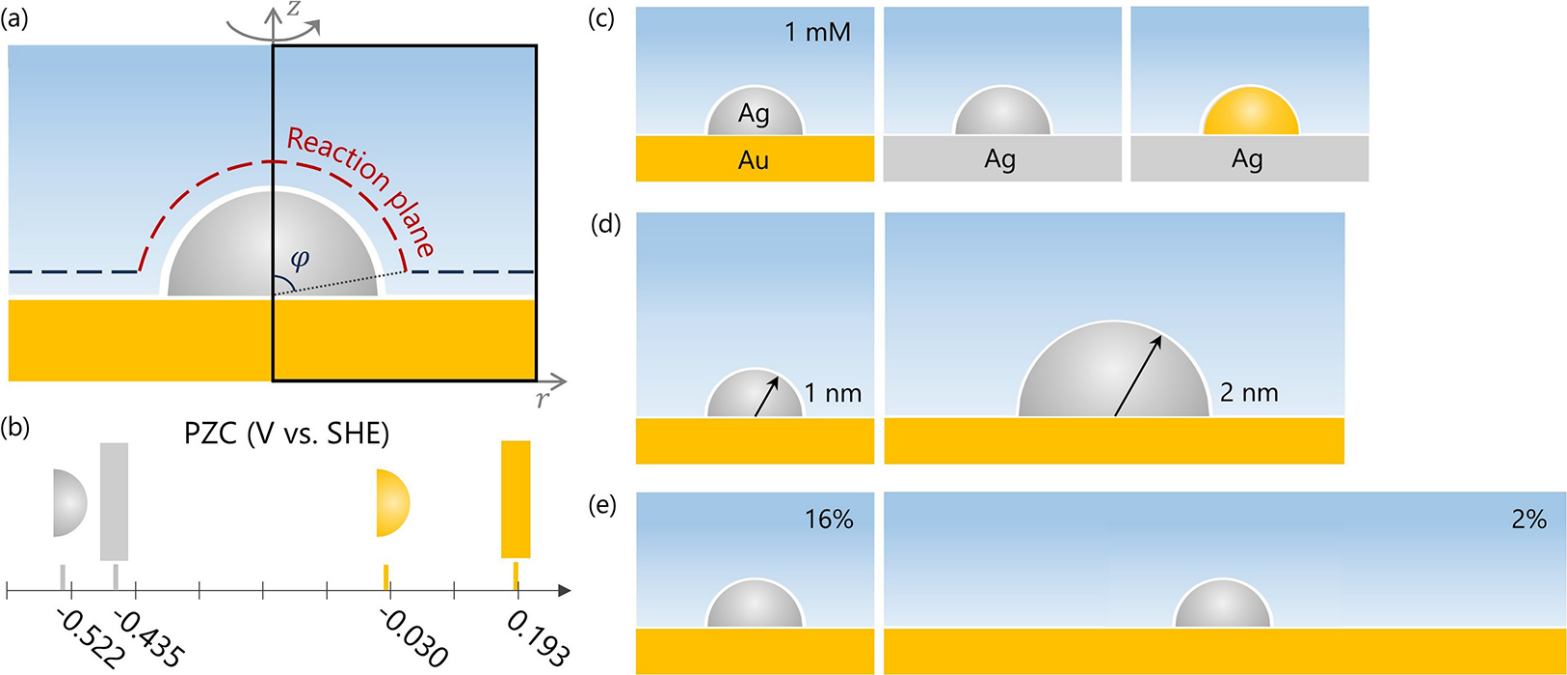
(a) Schematic representation of the supported NP system and the 2D modeling domain. (b) PZC of Ag NP, Ag support, Au NP, and Au support.^52–54^ (c–e) Tunable system parameters: (c) materials combination, (d) NP size, and (e) NCSF on support.

Figure 1b visualizes the PZCs of Au support, Ag support, Au NP and Ag NP. The PZC of Au support is 715 mV more positive than that of Ag NP. Additionally, the PZC of the NP is more negative than that of the corresponding planar surface, due to the nanoscale surface curvature.^68^ Figure 1c–e illustrates the key parameters available for adjustment in this study: support material, particle radius, and NCSF on the support, summarized in Table 1. Additionally, we examine the influence of the bulk ion concentration on the system behavior. For the reference case, we have used a Ag NP of 1 nm radius attached to a planar Au surface immersed in a 100 mM HClO_4_ electrolyte solution, with protons as the only cation species. The simulation cell of the reference case has a radius of 2.5 nm, implying a NCSF of 16%.

**Table 1:** Base Case Parameters and Their Variations

	Parameters	Base case	Variations				
1	Materials combination	Ag@Au	Ag@Ag			Au@Ag	
2	NP radius	1 nm	2 nm				
3	NP-covered support surface fraction (NCSF)	16%	2%				
4	Bulk proton concentration	100 mM	1 mM				
5	Electrode potential (V vs. SHE)	–0.522	–0.4	–0.3	–0.2	–0.093	0.193
	(V vs. RHE at pH = 3)	–0.345	–0.223	–0.123	–0.023	0.084	0.37

### Density Potential Functional Theory (DPFT) Approach

DPFT constitutes a self-consistent and efficient approach to treat electronic and ionic contributions to the interfacial charging behavior.^37,38^ Metal atomic-core charges are represented in this approach as a uniform background (so-called jellium).^55,56^ Minimizing the grand potential functional, Ω, of the system yields equilibrium distributions of key variables, including electric potential, *ϕ*, electron density, *n*_e_, and number densities of ionic species in the electrolyte solution (with subscripts c and a for cations and anions).

The expression for Ω is given by

1 $$\Omega =\int gd^{3}r=\int \left(f_{Q}+f_{C}+f_{\mathrm{int}}-n_{e}{\mathrm{\mu ̃}}_{e}-n_{c}{\mathrm{\mu ̃}}_{c}-n_{a}{\mathrm{\mu ̃}}_{a}\right)d^{3}r$$

where *g* denotes the volumetric density of Ω. The Helmholtz free energy encompasses three parts, namely the quantum contribution for the electron gas, *f*_Q_, the classical contribution for the charged species, *f*_C_, and the short-range interaction between electron gas and classical charged species, *f*_int_. Expressions for *f*_Q_, *f*_C_, and *f*_int_ are provided in the Supporting Information. The last three terms in eq 1, with ${\mathrm{\mu ̃}}_{l}\left(l=e,c,a\right)$ denoting electrochemical potentials, ensure that the interface system studied can freely exchange electrons and ions with corresponding reservoirs.

Applying the Euler–Lagrange equation with *ϕ* as the variational variable, $\frac{\partial g}{\partial \phi }-\nabla \cdot (\frac{\partial g}{\partial \left(\nabla \phi \right)})=0$ , yields the modified Poisson equation,^57^

$$-\nabla \cdot \left(\epsilon_{\mathrm{eff}}\nabla \phi \right)=e\left(n_{\mathrm{cc}}-n_{e}\right)+e\left(n_{c}-n_{a}\right)-\nabla \cdot \left[n_{s}\left|\mathrm{p⃗}\right|\frac{L\left(\beta \left|\mathrm{p⃗}\right|\left|{\mathrm{E⃗}}_{\mathrm{tot}}\right|\right)}{\left|{\mathrm{E⃗}}_{\mathrm{tot}}\right|}\mathrm{A⃗}\right]$$ 2

with L(x)=coth(x)−1/x being the Langevin function, *n*_cc_ denoting the charge density of metal cationic cores, *n*_s_ the number density of solvent molecules, ϵ_eff_ the effective dielectric constant, *p⃗* the dipole moment of solvent molecules, *E⃗*_tot_ the effective total field, *A⃗* the auxiliary field specific for a given electrode composition and surface crystal orientation, and *β* the inverse thermal energy. Expressions of *n*_c_, *n*_a_, *n*_s_, ϵ_eff_, *p⃗*, *A⃗,* and *E⃗*_tot_ are given in the Supporting Information.

For the distribution of electrons, following $\frac{\partial g}{\partial n_{e}}-\nabla \cdot (\frac{\partial g}{\partial \left(\nabla n_{e}\right)})=0$ , we derive

$$\nabla \cdot [\frac{\partial \left(t_{\mathrm{ni}}+u_{x}+u_{c}+p_{\mathrm{cc}}\right)}{\partial \nabla n_{e}}]=\frac{\partial \left(t_{\mathrm{ni}}+u_{x}+u_{c}+p_{\mathrm{cc}}\right)}{\partial n_{e}}-e\;\phi -{\mathrm{\mu ̃}}_{e}$$ 3

where *t*_ni_ is the kinetic energy of noninteracting (subscript “ni”) electrons, *u*_X_ and *u*_C_ are the exchange and correlation energies, and *p*_cc_ is the pseudopotential energy. Expressions of *t*_ni_, *u*_X_, *u*_C_, and *p*_cc_ are provided in the Supporting Information.

The controlling equations are solved in COMSOL Multiphysics to yield the equilibrium profiles of *n*_e_, *ϕ*, *n*_c_, and *n*_a_. Differentiation of different electrode materials is accomplished by an elaborate calibration process that yields the input parameters for the model. Specifically, the calibration process follows a systematic procedure, focusing in separate steps on: (i) electronic properties, (ii) dielectric properties, and (iii) ionic properties, using experimental data for work function, Φ,^58–61^ potentials of zero free charge, PZC,^52–54^ and differential capacitances, *C*_d_,^52,54^ of Ag and Au electrodes, respectively.^36^ This three-step procedure ensures that all three types of properties can be captured by a unique set of parameters. The Supporting Information details the calibration process.

### Effective Proton Concentration as a Descriptor for the Local Reaction Environment

On planar catalytic surfaces with crystalline order, the reactant concentration at the RP is spatially homogeneous. In such systems, the rate of proton-consuming electrochemical reactions, like the hydrogen evolution, oxygen and carbon dioxide reduction, or Pt dissolution, correlates positively with the local proton concentration at the RP.^62–66^ For example, platinum can dissolve via three major pathways: (i) direct anodic dissolution of metallic Pt, (ii) chemical dissolution of anodically formed PtO, and (iii) cathodic dissolution of PtO_2_ during oxide reduction.^63,67^ Among these, the latter two involve protons as reactants and therefore exhibit pronounced pH dependencies. Experiments have shown that the amount of dissolved Pt increases by more than 2 orders of magnitude with a 50-fold rise in acid concentration, confirming that proton concentration critically controls dissolution kinetics.^65^

However, in supported NP systems the situation is more complex. Firstly, the spatial distribution of the electrolyte is no longer uniform at the RP due to nanoscale curvature, heterogeneous surface structures, and catalyst–support interactions. Secondly, NP surfaces feature a range of crystallographic facets, including terraces, edges, and corners, each exhibiting distinct catalytic activity. While these atomistic features are critical, identifying the most catalytically active sites remains challenging and lies beyond the resolution of mean-field models.

Therefore, to characterize the LRE in a physically meaningful and computationally accessible way, we introduce a descriptor: the average proton concentration over the entire NP’s RP. This quantity provides a first-order approximation of proton availability at the active proportion of the interface and it enables comparison across different NP sizes, NP densities, support types, and operating conditions.

For proton-consuming reactions, this effective proton concentration is directly related to catalytic activity. It captures in highly condensed form the interplay among NP morphology, positions of the Fermi levels of NP and support materials, and ion concentration distributions, making it a valuable descriptor for understanding and predicting structure–property relationships in electrocatalytic NP systems.

Here, the RP is assumed to be located at the same position as the outer Helmholtz plane, which represents the closest approach of solvated ions. Depending on the size of the solvated ions, the RP is typically positioned at a distance *L*_RP_ of around 0.5 nm from the electrode surface, as indicated by the red dashed arc in Figure 1a. In the following, we will plot not only the two-dimensional distribution of proton concentration but also the concentration profile along the red dashed arc.

The effective proton concentration $\left(\overset{®}{c_{H^{+}}^{\mathrm{RP}}\;}\right)$ is defined as the average *c*_H^+^_ over the hemispherical RP around the NP, which can be calculated by a line integral of *c*_H^+^_ along the arc $\overset{͡}{\mathrm{RP}}$ , weighted by 2*πr* and divided by the area, *S*_RP_, of the RP,

4 $$\overset{®}{c_{H^{+}}^{\mathrm{RP}}\;}=\frac{\int_{\overset{͡}{\mathrm{RP}}}c_{H^{+}}\left(r,z\right)2\pi r\;ds}{S_{\mathrm{RP}}}$$

where

5 $$S_{\mathrm{RP}}=2\pi {\left(R_{\mathrm{cat}}+L_{\mathrm{RP}}\right)}^{2}\left(1-\cos\varphi \right)$$

with d*s* being the infinitesimal arc length element of $\overset{͡}{\mathrm{RP}}$ and $\varphi =\frac{\pi }{2}-\arcsin\frac{L_{\mathrm{RP}}}{R_{\mathrm{cat}}+L_{\mathrm{RP}}}$ .

## Results

### Effect of the Support Material

In an effort to elucidate the influence of support properties on the LRE, we compare Ag NP supported on Au and Ag surfaces, focusing on their effect on the effective proton concentration, $\overset{®}{c_{H^{+}}^{\mathrm{RP}}\;}$ . Figure 2a and c show the proton concentration distribution around the NP, while Figure 2b and d display 1D concentration profiles along $\overset{͡}{\mathrm{RP}}$ . The values of calculated $\overset{®}{c_{H^{+}}^{\mathrm{RP}}\;}$ of a system are indicated by the diamond marks on the corresponding curves in Figure 2b and d.

**Figure 2:**
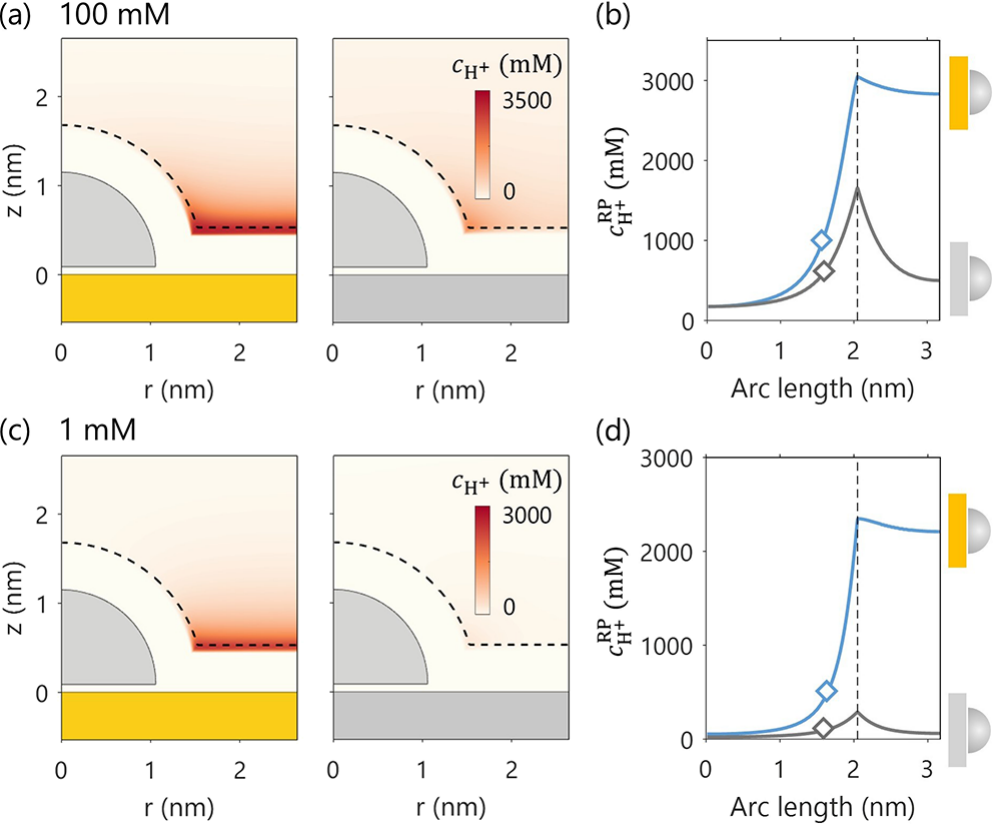
Effect of support material (Au vs Ag) on proton concentration distribution, *c*_H^+^_. Panels (a) and (c) show the 2D distribution of *c*_H^+^_ for bulk concentrations of 100 mM and 1 mM, respectively. Panels (b) and (d) display the corresponding 1D profiles of *c*_H^+^_ along the RP. The diamond marks in (b) and (d) denote the calculated $\overset{®}{c_{H^{+}}^{\mathrm{RP}}\;}$ . Vertical lines mark the boundary of the RP. The electrode potential is set to be the PZC of the hemispherical Ag NP, *E* = −0.522 V_SHE_The PZC of planar Ag and Au support are −0.435 V_SHE_ and 0.193 V_SHE_, respectively.

As a reference, we first investigate the base case of an Ag nanoparticle supported on Au. Since the Au support has a more positive PZC (0.193 V_SHE_) than Ag NP (−0.522 V_SHE_), at a given electrode potential, specifically, at the PZC of Ag NP, the Au surface is strongly negatively charged. This creates a negative potential region above the support surface, leading to proton accumulation. The close distance between NP and support renders the EDL structure around the NP to be strongly affected by the negative charges on the support surface. This EDL overlap effect is most pronounced in the vicinity of the NP–support interface, where the overlap is the strongest, as evidenced by the rising blue curve within the arc length range of 0 to 2 nm in Figure 2b and d.

Next, we evaluate how the proton concentration profile is altered when the support material is changed from Au to Ag. Given that the PZC of planar Ag support is −0.435 V_SHE_, the mismatch in the two PZC is smaller for the Ag NP–Ag support system than for the Ag NP–Au support system. As reflected by the lower gray curve compared to the blue curve in Figure 2b and d, we observe a lower proton concentration surrounding the Ag NP when it is supported on the Ag surface compared to the case on the Au surface. To summarize, the EDL overlap effect is especially prominent when there is a substantial difference in the PZC of NP and support.

The impact of the electrolyte concentration on the EDL overlap effect is studied by varying the electrolyte concentration from 100 mM to 1 mM. At 100 mM, $\overset{®}{c_{H^{+}}^{RP}\;}$ of Ag NPs supported on Au is roughly twice as large as on Ag, as shown by the diamond marks in Figure 2b. In contrast, at 1 mM, the difference increases markedly, from a 2-fold to a more than 5-fold variation. This suggests that the EDL overlap effect becomes more pronounced at lower electrolyte concentrations, as implied by the larger Debye length.

### Effect of NP Size

We turn the attention now to the influence of NP size on $\overset{®}{c_{H^{+}}^{\mathrm{RP}}\;}$ , which we analyze by comparing NPs with radii of 1 and 2 nm, while keeping the NCSF at 16%. It is well established that the work function and PZC of larger NPs are more positive than those of smaller NPs due to surface curvature effect.^68,69^ Indeed, our simulations show that an unsupported Ag NP with a 2 nm radius has a more positive PZC (−0.478 V_SHE_) compared to a 1 nm NP (−0.522 V_SHE_). As a result, at a given electrode potential, a larger unsupported NP would carry a more negative surface charge, leading to greater proton accumulation around it. However, this trend is reversed when the Ag NP is anchored on an Au support. As indicated by the diamond markers in Figure 3b and d, the average proton concentration is higher for smaller NP. This reversal is attributed to the EDL overlap effect, which is most prominent near the NP–support interface. For the larger NP, a significant portion of its surface lies farther from the support, reducing the influence of the support’s double layer on local proton concentration at the RP. This trend is observed under both electrolyte concentrations examined.

**Figure 3:**
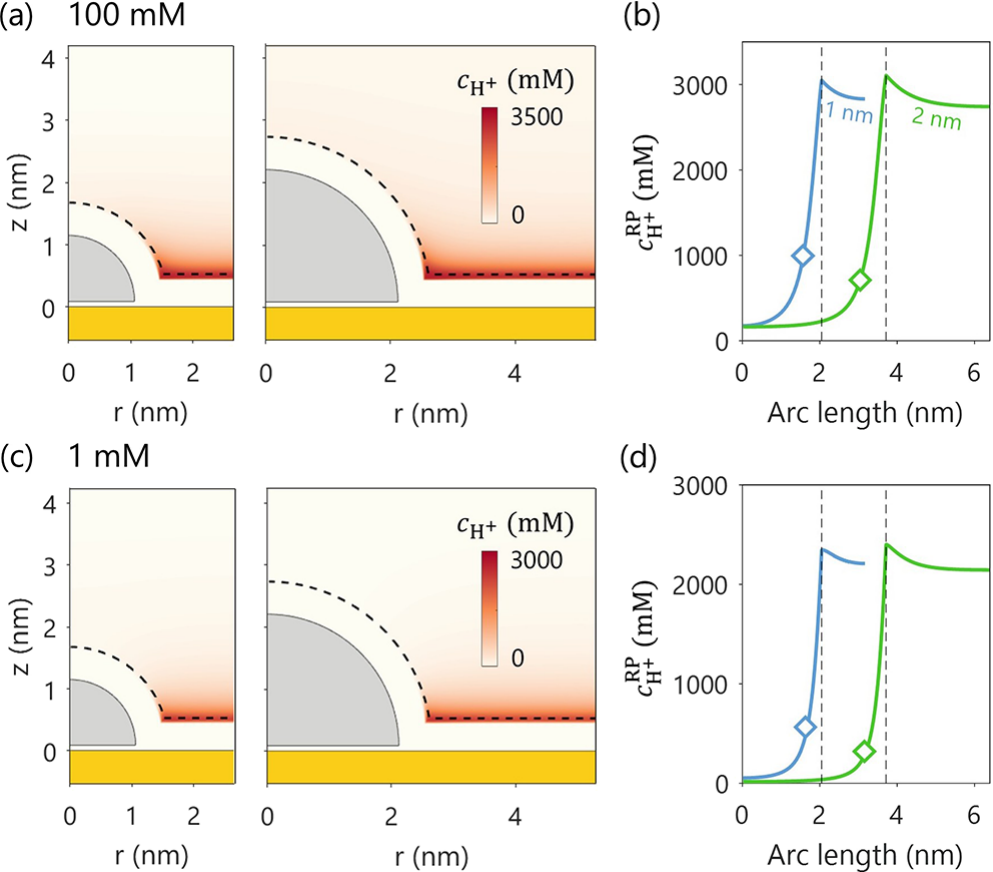
Effect of NP radius (1 nm vs 2 nm) on proton concentration distribution, *c*_H^+^_. Panels (a) and (c) show the 2D distribution of *c*_H^+^_ for bulk concentrations of 100 mM and 1 mM, respectively. Panels (b) and (d) display the corresponding 1D profiles of *c*_H^+^_ along the reaction plane. The diamond marks in (b) and (d) denote the calculated $\overset{®}{c_{H^{+}}^{\mathrm{RP}}\;}$ . The vertical lines mark the boundary of the RP. The electrode potential is set to be the PZC of the hemispherical Ag NP of 1 nm radius, *E* = −0.522 V_SHE_. The PZC of the 2 nm Ag NP and the planar Au support are −0.478 V_SHE_ and 0.193 V_SHE_, respectively.

### Effect of NCSF

Maintaining a fixed NP size while reducing the NCSF on the support corresponds to an increase in interparticle distance. Previous studies have shown that NP proximity significantly influences the catalytic activity of carbon-supported Pt NPs for oxygen reduction.^30,31^ At 0.9 V_RHE_, Pt NPs are negatively charged and thus protophilic, while the positively charged carbon support is protophobic. The overlap of their respective EDLs depletes the proton concentration near the Pt NPs. However, when the interparticle distance is reduced the EDL overlap is increasingly governed by the charging properties of neighboring Pt NPs, which amplifies the protophilic behavior and eventually dominates over the protophobic effect of the support. It was concluded that the NCSF or interparticle distance plays a crucial role in determining the accumulation and depletion of protons.

In the work presented here, we have revisited the effect of the NCSF. By increasing the simulation cell size, we reduce the NCSF from 16% to 2%, which increases the interparticle distance from 3 to 13 nm. As the Ag NP has a more negative PZC than a pcAu support, it bears more positive charges, and thus more protophobic. It was therefore expected that larger NCSF will repel protons more strongly, resulting lower proton concentration at the RP. However, the nearly identical *c*_H^+^_ profiles along the RP in Figure 4b show that the NCSF has a negligible effect on proton distribution. This counterintuitive result might be explained for the case of 100 mM bulk concentration, where the Debye length of 1 nm is smaller than both interparticle distances (3 and 13 nm), effectively screening electrostatic interactions between neighboring NPs. Surprisingly, even at a dilute solution of 1 mM concentration, where the Debye length is roughly 10 nm, there is still no discernible difference in $\overset{®}{c_{H^{+}}^{\mathrm{RP}}\;}$ between the two NCSF cases (Figure 4c,d). This observation highlights an important point: whether interparticle “communication” occurs is not governed by the bulk ion concentration or its associated Debye length, but rather by the local ion concentration in the space between neighboring NPs. This local ion concentration is governed by the surface charge density of the support. A more relevant length scale in this context is the Gouy–Chapman length, which is defined as $l_{\mathrm{GC}}=\frac{2\epsilon k_{B}T}{e\left|\sigma \right|}$ , where ϵ is the permittivity of the dielectric medium, *σ* the surface charge density, *k*_B_ Boltzmann constant, *T* temperature, and *e* elementary charge. The Gouy–Chapman length is inversely proportional to *σ*.^70^

**Figure 4:**
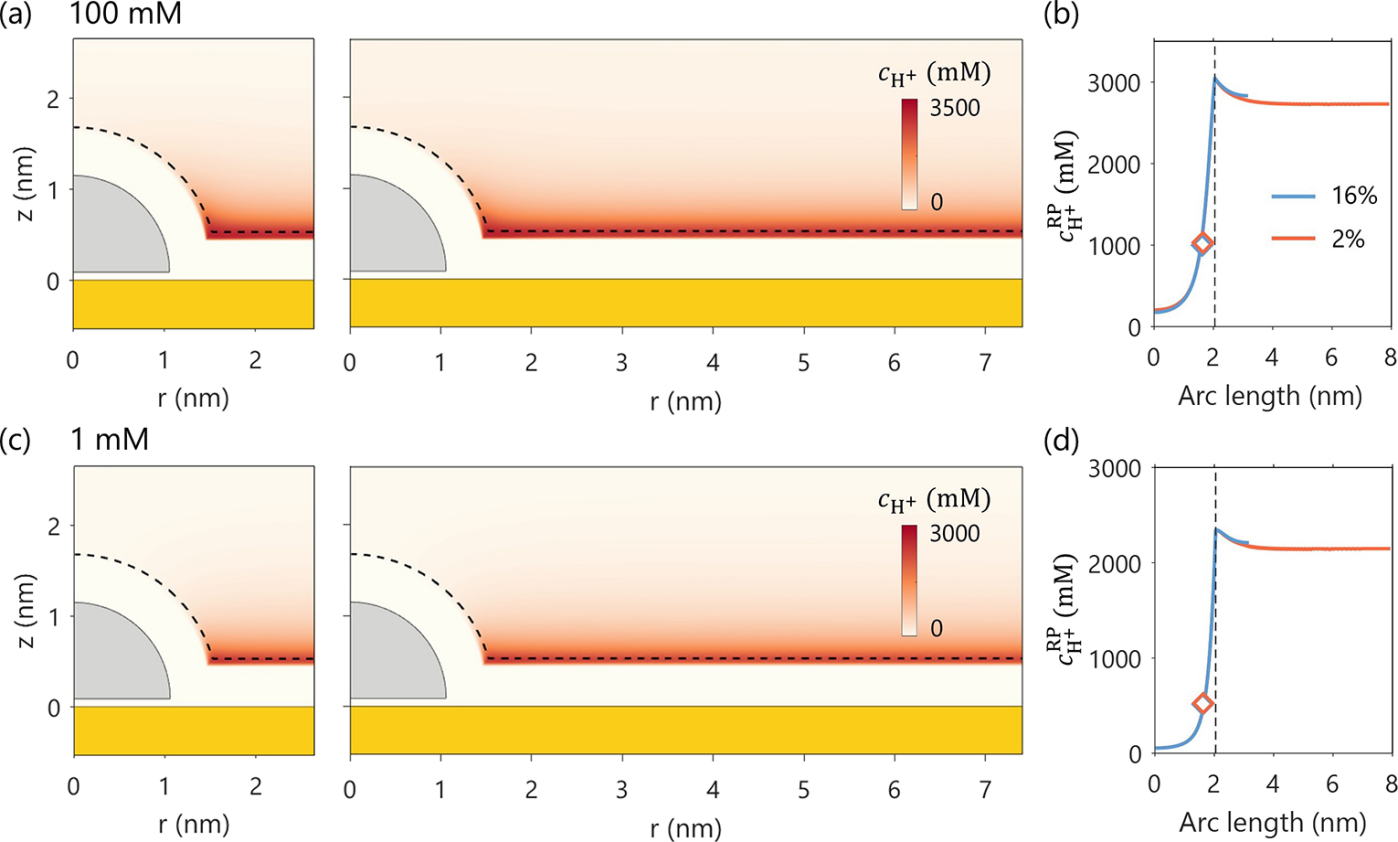
Effect of the NCSF (16% vs 2%) on proton concentration distribution, *c*_H^+^_. Panels (a) and (c) show the 2D distribution of *c*_H^+^_ for bulk concentrations of 100 mM and 1 mM, respectively. Panels (b) and (d) display the corresponding 1D profiles of *c*_H^+^_ along the reaction plane. The diamond symbols in (b) and (d) denote the calculated $\overset{®}{c_{H^{+}}^{\mathrm{RP}}\;}$ . The vertical lines mark the end of the RP. The electrode potential is set to be the PZC of the hemispherical Ag NP, *E* = −0.522 V_SHE_. The PZC of planar Au support is 0.193 V_SHE_.

At an applied potential of −0.522 V_SHE_, which is much more negative than the PZC of the Au support (0.193 V_SHE_), *σ* reaches 10 μC/cm^2^. This corresponds to a Gouy–Chapman length of 0.36 nm, much smaller than the interparticle distances in both cases (3 and 13 nm). As a result, the NPs are electrostatically isolated by the highly concentrated region near the support, explaining the negligible variation in $\overset{®}{c_{H^{+}}^{\mathrm{RP}}\;}$ with NP proximity.

The strong cation accumulation near the support surface is caused by the electrode potential being significantly more negative than the PZC of the support material. As this ion accumulation can be reduced by tuning the electrode potential toward more positive values, a natural question then arises: will the proximity effect emerge if fewer ions are present in the space between neighboring NPs?

Figure 5 presents the 2D distribution and 1D profile of proton concentration for a series of increasingly positive *E*. Figure 6 plots $\overset{®}{c_{H^{+}}^{\mathrm{RP}}\;}$ as a function of *E*, shown in both linear and logarithm scales. At strongly negative potentials, such as 0.522 V_SHE_ and −0.4 V_SHE_, no significant difference in $\overset{®}{c_{H^{+}}^{\mathrm{RP}}\;}$ is observed between the two particle densities. However, as *E* becomes more positive, at −0.3 V_SHE_ and −0.2 V_SHE_, a modest difference in $\overset{®}{c_{H^{+}}^{\mathrm{RP}}\;}$ of approximately 10 mM appears. At even more positive potentials, such as −0.093 V_SHE_ and 0.193 V_SHE_, this difference vanishes again, as protons are strongly repelled from the NP surfaces under these conditions.

**Figure 5:**
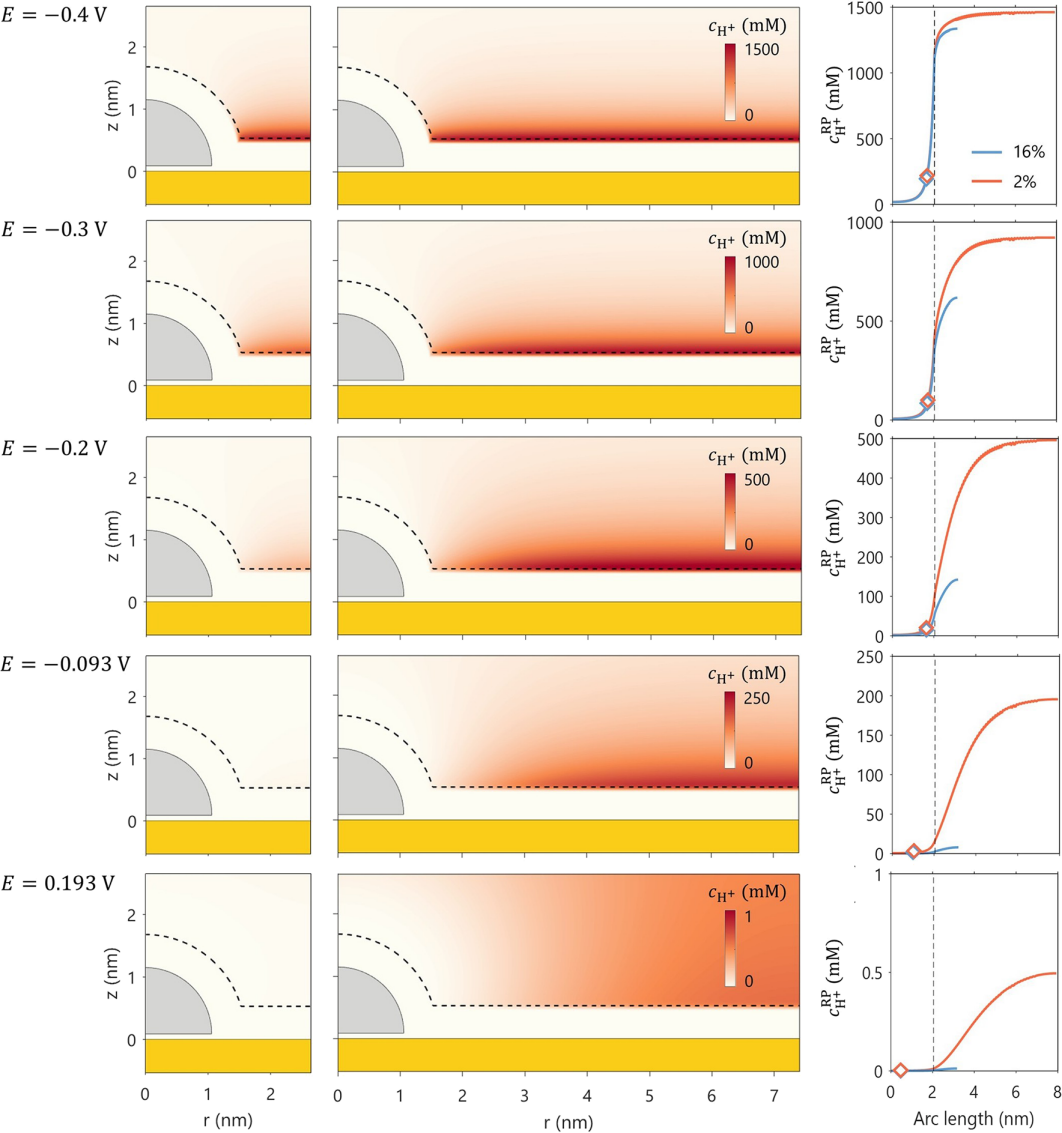
2D distribution of *c*_H^+^_ for bulk concentrations of 1 mM and the corresponding 1D profiles of *c*_H^+^_ profiles along the reaction plane for a series of electrode potentials as specified in the figure. The diamond symbols in the line graph denote the calculated $\overset{®}{c_{H^{+}}^{\mathrm{RP}}\;}$ . The vertical lines mark the end of the RP.

**Figure 6:**
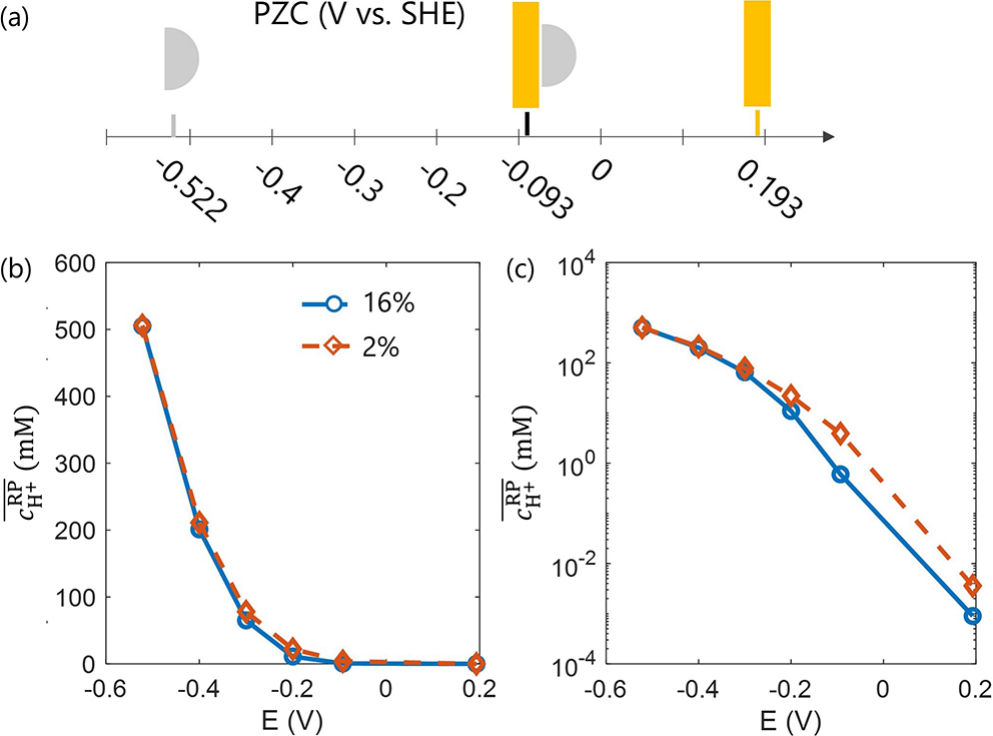
(a) A series of electrode potentials applied to the Au supported Ag NP system. (b) and (c) show $\overset{®}{c_{H^{+}}^{\mathrm{RP}}\;}$ as a function of electrode potentials (V vs. SHE) in linear and logarithmic scales.

Figures 5 and 6 reveal that, even though ion accumulation near the support surface is alleviated as *E* approaches the PZC of the support, protons are nonetheless repelled from the NP surface at very positive electrode potentials, and no significant particle proximity effect is observed. One would naturally ask: since anions will not be repelled from the NP, could their concentration be influenced by variations in NP proximity? As demonstrated in Figure 7, the answer is affirmative. While the proximity effect remains small in the 100 mM solution, it becomes noticeably more pronounced in the 1 mM case.

**Figure 7:**
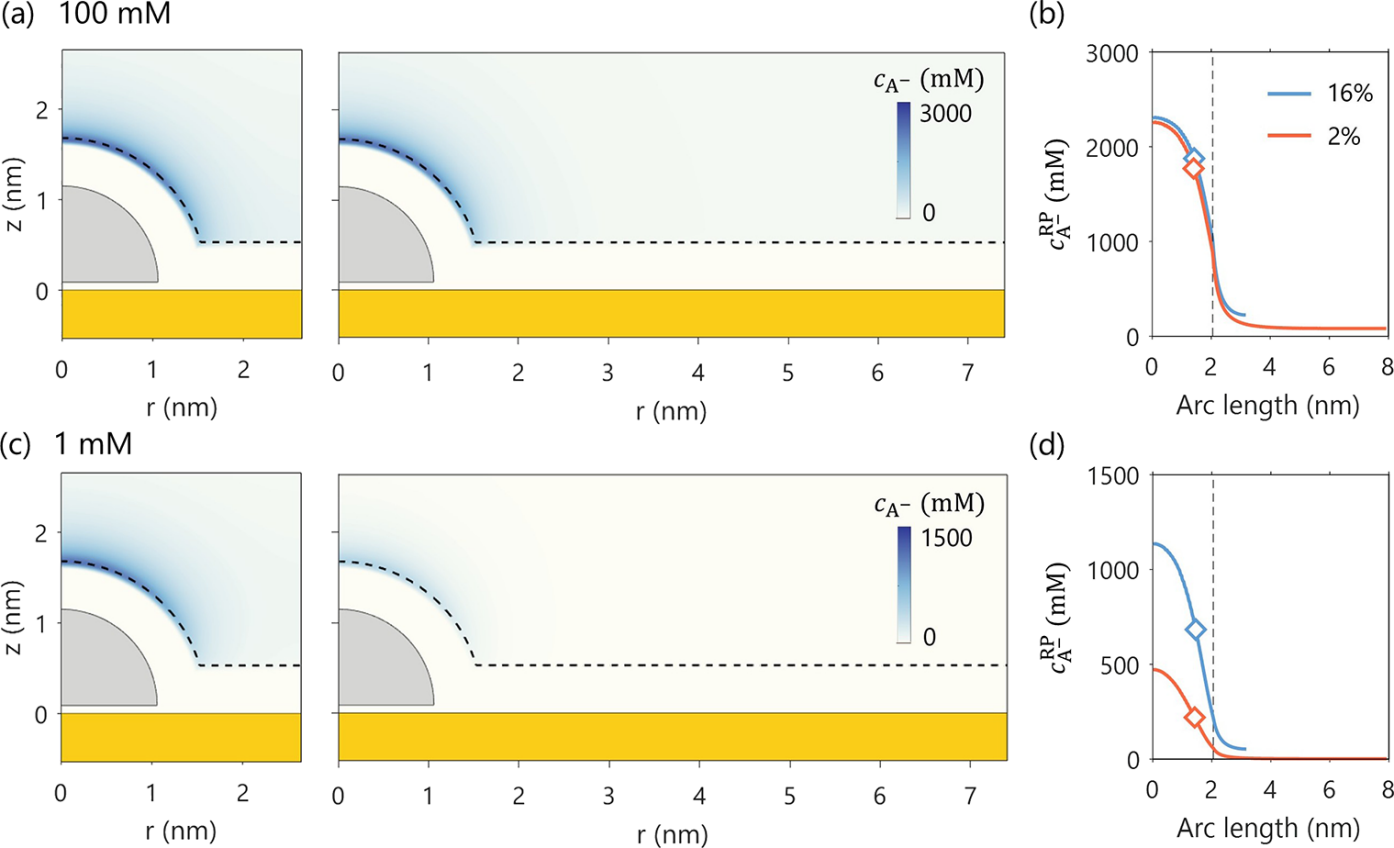
Effect of NCSF (16% vs 2%) on anion concentration distribution, $c_{A^{-}}$ . Panels (a) and (c) show the 2D distribution of $c_{A^{-}}$ for bulk concentrations of 100 mM and 1 mM, respectively. Panels (b) and (d) display the corresponding 1D profiles of $c_{A^{-}}$ along the reaction plane. The diamond symbols in (b) and (d) denote the calculated $\overset{®}{c_{A^{-}}^{\mathrm{RP}}\;}$ . The vertical lines mark the end of the RP. *E* = 0.193 V_SHE_ The electrode potential is set to be the PZC of planar Au support, *E* = −0.193 V_SHE_. The PZC of hemispherical Ag NP is −0.435 V_SHE_.

Above presented results and discussions can be subsumed into three main criteria that must be met for a pronounced NCSF (proximity) effect to emerge: (i) low bulk concentration in the solution, (ii) electrode potential close to the PZC of the support material, and (iii) a specific sequence of the PZC of the catalyst NP and the support such that the ions of interest are significantly more strongly attracted to the NP compared to the support. For proton-consuming reactions, this implies that the PZC of the catalyst should be more positive than that of the support. To test this hypothesis, the materials of NP and support are reversed, viz., a Au NP supported on Ag, as illustrated in Figure 8. With the electrode potential set to be the PZC of Ag support (−0.435 V_SHE_), the 1 mM solution shows a clear proximity effect in proton concentration, validating our hypothesis.

**Figure 8:**
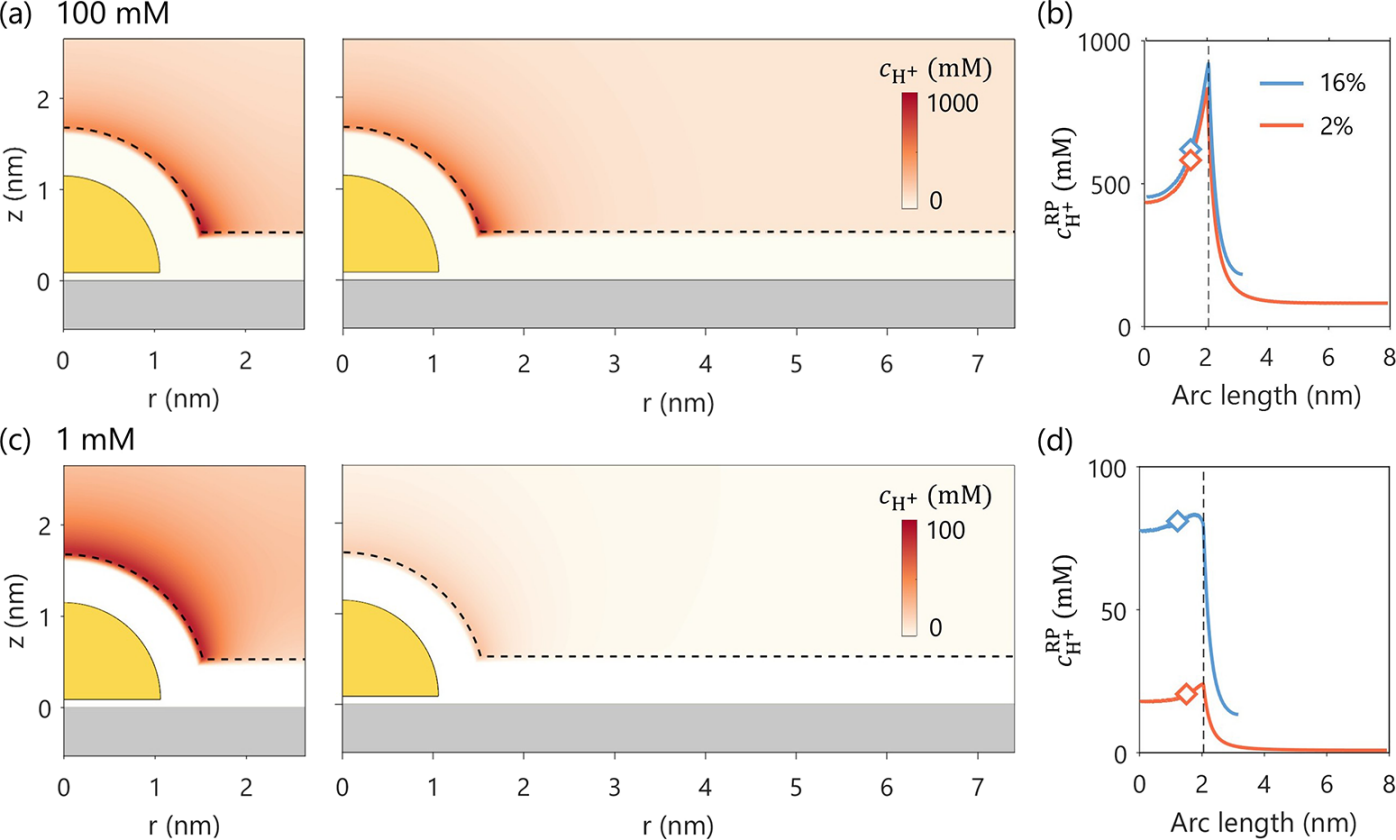
Effect of NCSF (16% vs 2%) on proton concentration distribution, *c*_H^+^_. Panels (a) and (c) show the 2D distribution of *c*_H^+^_ for bulk concentrations of 100 mM and 1 mM, respectively. Panels (b) and (d) display the corresponding 1D profiles of *c*_H^+^_ along the reaction plane. The diamond symbols in (b) and (d) denote the calculated $\overset{®}{c_{H^{+}}^{\mathrm{RP}}\;}$ . The vertical lines mark the end of the RP. *E* = −0.435 V_SHE_. The PZC for Au NP and Ag support are −0.03 V_SHE_ and −0.435 V_SHE_, respectively.

## Discussion

In the preceding section, we have analyzed how support material, NP size, NCSF, and electrode potential affect the LRE by monitoring the proposed LRE descriptor, namely the average proton concentration over the RP. The specific value of the LRE descriptor lies in its independence of parameters that are specific to reactions. In an attempt to further elucidate its impact on reaction rates, a simple Butler–Volmer-type kinetic model is built. The surface-area-specific activity is expressed as the average kinetic current density over the area of the reaction plane enveloping the particle,

6 $$\mathrm{j̅}=\frac{\int_{\overset{͡}{\mathrm{RP}}}j\left(r,z\right)2\pi r\;ds}{S_{\mathrm{RP}}}$$

where the spatially varying current density is given by the Butler–Volmer equation accounting for the Frumkin effect,

7 $$j\left(r,z\right)=j^{0}{\left(\frac{c_{H^{+}}^{\mathrm{RP}}\left(r,z\right)}{c_{H^{+}}^{b}}\right)}^{\gamma_{H^{+}}}\exp\left(-\frac{\alpha e\left(E-\Delta \phi_{\mathrm{RP}}\left(r,z\right)-E^{\mathrm{eq}}\right)}{k_{B}T}\right)$$

with *j* ^0^ being the exchange current density, γ_H^+^_ the reaction order of protons, $c_{H^{+}}^{\mathrm{RP}}$ and $c_{H^{+}}^{b}$ the proton concentrations at the RP and in the bulk solution, α the transfer coefficient, Δ*ϕ*_RP_ the deviation of the electrostatic potential at the RP from the value in solution bulk, and *E* ^eq^ the equilibrium potential. Note in passing that *j* ^0^, $c_{H^{+}}^{b}$ and *E* ^eq^ are interrelated: $c_{H^{+}}^{b}$ corresponds to the bulk concentration at which *j* ^0^ is measured, and *E* ^eq^ refers to the equilibrium potential at a solution of that same bulk concentration.

Separating location-dependent and independent terms in eq 7 yields,

8 $$j=j^{0}{c_{H^{+}}^{b}}^{-\gamma_{H^{+}}}\exp\left(-\frac{\alpha e\left(E-E^{\mathrm{eq}}\right)}{k_{B}T}\right){\left(c_{H^{+}}^{\mathrm{RP}}\left(r,z\right)\right)}^{\gamma_{H^{+}}}\exp{\left(\frac{e\Delta \phi_{\mathrm{RP}}\left(r,z\right)}{k_{B}T}\right)}^{\alpha }$$

The first three factors on the right-hand side of eq 8 are spatially invariant, while the last two factors incorporate the LRE effects. In addition to $c_{H^{+}}^{\mathrm{RP}}$ , Δ*ϕ*_RP_ also modulates the reaction rate through the exponential term of driving force, indicating that an LRE descriptor that is solely based on proton concentration cannot fully capture the LRE effects. To complement it, we denote the exponential factor by the symbol Λ,

9 $$\Lambda \left(r,z\right)=\exp\left(\frac{e\Delta \phi \left(r,z\right)}{k_{B}T}\right)$$

and introduce its area-averaged value over the RP as a complementary LRE descriptor, providing a mean-field-type measure of the spatial variations in the electrostatic potential,

10 $$\overset{®}{\Lambda_{\mathrm{RP}}\;}=\frac{\int_{\overset{͡}{\mathrm{RP}}}\Lambda_{\mathrm{RP}}2\pi r\;ds}{S_{\mathrm{RP}}}=\frac{\int_{\overset{͡}{\mathrm{RP}}}\exp\left(\frac{e\Delta \phi_{\mathrm{RP}}\left(r,z\right)}{k_{B}T}\right)2\pi r\;ds}{S_{\mathrm{RP}}}$$

Note that $\overset{®}{\Lambda_{\mathrm{RP}}\;}$ is likewise reaction-agnostic; as an LRE descriptor, it reflects only the properties of the LRE and not the kinetic specifics of any reaction.

The surface-area-specific activity, viz., the area-averaged kinetic current density, can be rewritten by substituting eqs 8 and 9 into eq 6,

11 $$\mathrm{j̅}=j^{0}{c_{H^{+}}^{b}}^{-\gamma_{H^{+}}}\exp\left(-\frac{\alpha e\left(E-E^{\mathrm{eq}}\right)}{k_{B}T}\right)\frac{\int_{\overset{͡}{\mathrm{RP}}}{\left(c_{H^{+}}^{\mathrm{RP}}\left(r,z\right)\right)}^{\gamma_{H^{+}}}{\left(\Lambda_{\mathrm{RP}}\left(r,z\right)\right)}^{\alpha }2\pi r\;ds}{S_{\mathrm{RP}}}$$

Making use of the two defined LRE descriptors, an approximate expression of the kinetic current density can be written by replacing the spatially varying $c_{H^{+}}^{\mathrm{RP}}\left(r,z\right)$ and $\Lambda_{\mathrm{RP}}\left(r,z\right)$ in eq 11 with their average values, $\overset{®}{c_{H^{+}}^{\mathrm{RP}}\;}$ and $\overset{®}{\Lambda_{\mathrm{RP}}\;}$ ,

12 $$\begin{array}{c} \overset{®}{j_{\mathrm{ap}}\;}=j^{0}{c_{H^{+}}^{b}}^{-\gamma_{H^{+}}}\exp\left(-\frac{\alpha e\left(E-E^{\mathrm{eq}}\right)}{k_{B}T}\right)\frac{\int_{\overset{͡}{\mathrm{RP}}}{\left(\overset{®}{c_{H^{+}}^{\mathrm{RP}}\;}\right)}^{\gamma_{H^{+}}}{\left(\overset{®}{\Lambda_{\mathrm{RP}}\;}\right)}^{\alpha }2\pi r\;ds}{S_{\mathrm{RP}}},\\ \overset{®}{j_{\mathrm{ap}}\;}=j^{0}{c_{H^{+}}^{b}}^{-\gamma_{H^{+}}}\exp\left(-\frac{\alpha e\left(E-E^{\mathrm{eq}}\right)}{k_{B}T}\right){\left(\overset{®}{c_{H^{+}}^{\mathrm{RP}}\;}\right)}^{\gamma_{H^{+}}}{\left(\overset{®}{\Lambda_{\mathrm{RP}}\;}\right)}^{\alpha } \end{array}$$

Although eq 12 is not strictly equivalent to eq 11 (since the product of averages is not equal to the average of the product), it turns out to be a reasonable approximation for many cases (for a detailed discussion, vide infra). The practical value of eq 12 is that it enables the definition of an activity descriptor of the form ${\left(\overset{®}{c_{H^{+}}^{\mathrm{RP}}\;}\right)}^{\gamma_{H^{+}}}{\left(\overset{®}{\Lambda_{\mathrm{RP}}\;}\right)}^{\alpha }$ that is a combination of two reaction-agnostic LRE descriptors, $\overset{®}{c_{H^{+}}^{\mathrm{RP}}\;}$ and $\overset{®}{\Lambda_{\mathrm{RP}}\;}$ , with the specifics of a particular reaction entered through the kinetic parameters $\gamma_{H^{+}}$ and *α*.

We expect this representation to be of practical utility since it allows the impact of the catalyst–support combination and nanoscale configuration to be accounted for explicitly through the LRE descriptors. They can therefore be tabulated or computed in advance (as a first step) for a given supported NP system. In a separate step, researchers interested in a particular reaction can combine these two values with reaction-specific parameters $\gamma_{H^{+}}$ and *α* to determine ${\left(\overset{®}{c_{H^{+}}^{\mathrm{RP}}\;}\right)}^{\gamma_{H^{+}}}{\left(\overset{®}{\Lambda_{\mathrm{RP}}\;}\right)}^{\alpha }$ for an evaluation or prediction of activity trends.

Of course, for each specific reaction one could alternatively use the average Frumkin factor, $\frac{\int_{\overset{͡}{\mathrm{RP}}}\exp\left(\frac{-\left(\gamma_{H^{+}}-\alpha \right)e\Delta \phi_{\mathrm{RP}}}{k_{B}T}\right)2\pi r\;ds}{S_{\mathrm{RP}}}$ , derived by approximating $c_{H^{+}}^{\mathrm{RP}}$ in eq 11 with Boltzmann distribution, as an activity descriptor. However, this requires recalculating the full spatial distributions of $c_{H^{+}}^{\mathrm{RP}}\left(r,z\right)$ and Λ_RP_(*r*,*z*). In contrast, our proposed form ${\left(\overset{®}{c_{H^{+}}^{\mathrm{RP}}\;}\right)}^{\gamma_{H^{+}}}{\left(\overset{®}{\Lambda_{\mathrm{RP}}\;}\right)}^{\alpha }$ provides a more efficient and transferable means of screening material combinations and configurations for reactions of interest, without the need for solving the spatial averages again.

We now proceed to illustrate the formal stepwise analysis, proposed above, with an example. Since the exchange current density of HER on Ag is 2 orders of magnitude lower than that of Au,^71–73^ we study Au NP supported on Ag surface and neglect the HER contribution from the Ag support. Figure 9a and b shows the 2D distributions of Λ as well as the Λ_RP_ along the RP for NCSF of 16%, 10%, 6%, and 2%. Since the Au NP is more negatively charged than the Ag support, closer interparticle distance gives more negative Φ_RP_, resulting in lower Λ_RP_.

Using eq 11, we calculate the surface-area specific activity for these NCSF, examining two representative parameter sets: (i) $\gamma_{H^{+}}=1$ and *α* = 0.5 (a reasonable combination) and (ii) $\gamma_{H^{+}}=\alpha =0.5$ (a less common but physically possible combination). To probe the quantitative relationship between LRE effects and reaction rate, the normalized quantities $\overset{®}{c_{H^{+}}^{\mathrm{RP}}\;}$ , $\overset{®}{\Lambda_{\mathrm{RP}}\;}$ and *j̅* are plotted versus NCSF in Figure 9c. Normalization was achieved by dividing each series by its minimal value, i.e., *j̅*/min­(*j̅*), $\overset{®}{c_{H^{+}}^{\mathrm{RP}}\;}/\min\left(\overset{®}{c_{H^{+}}^{\mathrm{RP}}\;}\right)$ , and $\overset{®}{\Lambda_{\mathrm{RP}}\;}/\min\left(\overset{®}{\Lambda_{\mathrm{RP}}\;}\right)$ .

For the case of $\gamma_{H^{+}}=1$ and *α* = 0.5, both *j̅* and $\overset{®}{c_{H^{+}}\;}$ increase with NCSF, whereas $\overset{®}{\Lambda_{\mathrm{RP}}\;}$ decreases, see Figure 9c. Recalling eq 12, when the two descriptors are combined as ${\left(\overset{®}{c_{H^{+}}^{\mathrm{RP}}\;}\right)}^{\gamma_{H^{+}}}{\left(\overset{®}{\Lambda_{\mathrm{RP}}\;}\right)}^{\alpha }$ , the resulting trend aligns closely with that of *j̅*, showing an approximately linear correlation, as can be seen Figure 9d. Indeed, the approximation, eq 12, reproduces the fully resolved current density, eq 11, with good agreement, as shown in Figure 9e.

The discrepancy between *j̅* and $\overset{®}{j_{\mathrm{ap}}\;}$ could be larger when the spatial variations of the electrostatic potential and proton concentration over the RP are larger, which in turn are primarily controlled by the PZC difference between the NP and the support. In our Ag-supported Au NP system, this PZC difference is already substantial (≈0.4 V), and most practical catalyst–support combinations are expected to exhibits differences in PZC of similar or smaller magnitude.^74,75^ Thus, the approximation appears justified in more general terms. It is worth noting that Au-supported Ag NP system (Figure 4d) has larger PZC difference (0.715 V) and therefore exhibits larger variations in proton concentration over the RP than Ag supported Au NP system (Figure 8d). Therefore, the activity descriptor should be more suitable for the former case than the latter one.

For the case $\gamma_{H^{+}}=\alpha =0.5$ (Figure 9f), *j̅* remains nearly constant with NCSF and neither $\overset{®}{c_{H^{+}}^{\mathrm{RP}}\;}$ nor $\overset{®}{\Lambda_{\mathrm{RP}}\;}$ alone captures this behavior; only their combined form ${\left(\overset{®}{c_{H^{+}}^{\mathrm{RP}}\;}\right)}^{\gamma_{H^{+}}}{\left(\overset{®}{\Lambda_{\mathrm{RP}}\;}\right)}^{\alpha }$ reproduces the correct trend. The underlying reason is, to a first approximation, $c_{H^{+}}^{\mathrm{RP}}$ follows a Boltzmann distribution, i.e., $c_{H^{+}}^{\mathrm{RP}}\propto \exp\left(-e\Delta \phi_{\mathrm{RP}}/k_{B}T\right)$ ; thus, when $\gamma_{H^{+}}=\alpha$ the potential dependence cancels exactly in the exponential term.

It is worth noting that the effective proton concentration, while not directly measurable experimentally, serves as a computational descriptor of the LRE. Similar to widely used descriptors in catalysis, such as the d-band center,^76^ this quantity provides a measure that connects computed properties to observable electrochemical behavior. By averaging over the RP, it captures the heterogeneity of the double layer around supported NPs and allows for systematic comparison across different materials, particle sizes, and electrode configurations. Therefore, even though it is model-specific, the effective proton concentration offers a practical and physically meaningful metric for understanding and rationally designing NP-based electrocatalysts.

Traditionally, the design of supported electrocatalysts often focuses on optimizing electronic structure, morphology, or metal–support interactions. Our findings show that electrochemical local reaction environment and EDL overlap effects should also be considered. Specifically, our descriptor-based analysis shed light on several new strategies:

(1) Selecting a suitable support material: choosing a support material with a PZC that is significantly different from that of the catalyst NP can be used to tailor local proton availability through EDL overlap.
(2) Tuning the NCSF or interparticle distance: optimal NCSF can be guided by maximizing ${\left(\overset{®}{c_{H^{+}}^{\mathrm{RP}}\;}\right)}^{\gamma_{H^{+}}}{\left(\overset{®}{\Lambda_{\mathrm{RP}}\;}\right)}^{\alpha }$ , rather than purely maximizing electrochemically active surface area.
(3) Electrolyte concentration: adjusting bulk ion concentration or supporting electrolyte composition can be used to amplify or dampen the EDL overlap effect.

**Figure 9:**
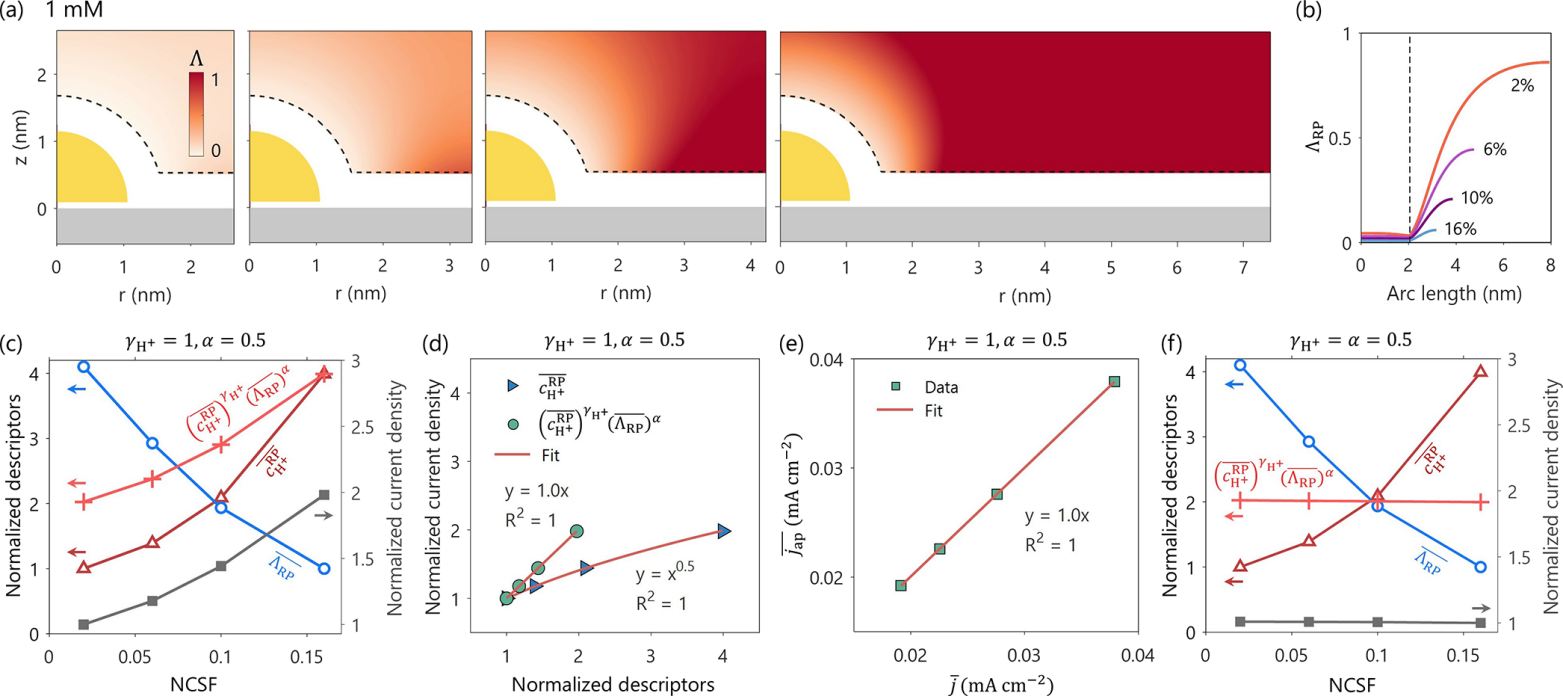
(a) 2D distribution of Λ for NCSF of 16%, 10%, 6%, and 2%. The bulk proton concentration is 1 mM. (b) Corresponding 1D profiles of Λ_RP_ along the reaction plane. The PZC for Au NP and Ag support are −0.03 V_SHE_ and −0.435 V_SHE_, respectively. *E* = −0.435 V_SHE_. (c)–(f) Quantitative relationships between the LRE descriptors and reaction rate of the hydrogen evolution reaction. Panel (c) and (f) show the normalized descriptors and current densities as functions of NCSF for the case of $\gamma_{H^{+}}=1$ , *α* = 0.5, and $\gamma_{H^{+}}=\alpha =0.5$ , respectively. (d) Correlation between the normalized descriptors and normalized current density. (e) Correlation between the averaged current density *j̅* and its approximation $\overset{®}{j_{\mathrm{ap}}\;}$ . The exchange current density is taken as 10^–3.5^ mA cm^–2^ in 100 mM HCl solution, and *α* = 0.5.^71^

## Conclusions

In this study, we have presented results obtained with a recently developed modeling approach for electrochemical interfaces to investigate the local reaction environment around supported electrocatalyst nanoparticles. The average ion concentration over the reaction plane is proposed as a descriptor for the local reaction environment in such systems. The presented findings reveal that the double layer overlap between nanoparticle and support plays an important role in shaping local ion density distributions, particularly near the nanoparticle–support contact. The impact of various key parameters, including support material, NP size and NCSF, electrolyte concentration, and electrode potential, on the local reaction environment has been explored.

(1) Support material: the reactant ion concentration at the reaction plane can be markedly raised by a support material that attracts reactants. For proton-consuming reactions, the support material should possess a more positive PZC than the catalyst NP.
(2) NP size: smaller NPs exhibit a stronger EDL overlap effect due to closer interactions with the support surface, resulting in higher reactant concentrations; this effect weakens with increasing NP size, because the RP of larger particles locates farther away from the support surface.
(3) NCSF or NP proximity: the interparticle distance has a minimal effect on proton distribution, not only in high ionic strength solutions (100 mM), but also at low ionic strength (1 mM). This seemingly counterintuitive observation shows that the local ion concentrations and the corresponding Gouy–Chapman length, rather than the bulk Debye length, govern the strength of interparticle EDL overlap. Our study identifies three prerequisites for a pronounced proximity effect: low bulk electrolyte concentration, electrode potential near the PZC of the support, and a PZC sequence that favors attraction of ion species of interest, *viz*. either cations or anions, to the NP. In reality, these conditions are rarely met in real electrochemical environments.
(4) Electrode potential: tuning the electrode potential closer to the PZC of the support can mitigate ion accumulation on the support surface, allowing the EDL overlap effect to be more visible.
(5) Bulk ion concentration: a reduction in bulk ion concentration amplifies the EDL overlap effect. However, for the NCSF/proximity effect to become noticeable, low bulk ion concentration serves as a necessary but not sufficient condition. The relative position of the electrode potential, the PZC of the catalyst NP, and the PZC of the support material play an important role in governing the NCSF/proximity effect.

To accurately capture the connection between the local reaction environment and electrochemical activity, we have defined a complementary descriptor that incorporates variations in local electrostatic potential. This descriptor accounts for the impact of variations in the local potential at the reaction plane on the driving force for electrochemical reactions. By combining this descriptor with the effective proton concentration, a unified activity descriptor is obtained. This combined descriptor incorporates the impact of the reaction order with respect to the proton concentration and the transfer coefficient in the kinetic rate equation, constituting a simplified variant of the widely known Frumkin effect. It quantitatively reproduces current density trends, thereby linking the peculiar microscopic reaction environment of supported nanoparticle systems with observable electrochemical kinetics.

## Supplementary Material



## Data Availability

All data are available from the author upon reasonable request.

## Notes

Equations of density potential functional theory (DPFT); three-step parameter calibration; equilibrium distance between nanoparticle and support (DOCX)
