# Supplementary material for: Effective Ion Concentration as a Descriptor for the Local Reaction Environment at Nanoparticle-Based Electrocatalysts
Source: ACS Catal. 2026 Feb 10;16(4):3175–87. doi: 10.1021/acscatal.5c06754 (PMC12930386; doi:10.1021/acscatal.5c06754)
Supplement: Supplementary file 1 [file cs5c06754_si_001.docx]

| **Effective Ion Concentration as a Descriptor for the Local Reaction Environment at Nanoparticle-Based Electrocatalysts** |
| --- |

Supporting Information

Yufan Zhang^1,2^, Tobias Binninger^1^, Jun Huang^1,2^, Michael H. Eikerling^1,2^

*1 Theory and Computation of Energy Materials (IET-3), Institute of Energy Technologies, Forschungszentrum Jülich GmbH, 52425 Jülich, Germany*

*2 Chair of Theory and Computation of Energy Materials, Faculty of Georesources and Materials Engineering, RWTH Aachen University, 52062 Aachen, Germany*

** Corresponding author: m.eikerling@fz-juelich.de*

**Contents**

[Equations of density potential functional theory (DPFT) 2](#_Toc178970608)

[Three-step parameter calibration 9](#_Toc178970609)

[Equilibrium distance between nanoparticle and support 12](#_Toc178970610)

[References 13](#_Toc178970611)

## Equations of density potential functional theory (DPFT)

The DPFT methodology, used here, has been developed to study both the electronic and ionic degrees of freedom in the electrical double layer (EDL) [1–4]. Initially focused on planar electrodes, we extended the framework to apply to supported electrocatalyst nanoparticles (NP) in our recent publication [5]. In the present study, we adopt the latest version of equations from Ref. [5]. For completeness, we briefly reproduce the key equations and explain the modifications for supported NP systems.

#### Thermodynamic potential for the grand canonical ensemble

Minimization of the grand potential functional ($\Omega$) of the system gives the equilibrium profiles of variables, namely, electric potential ($\phi$), electron density ($n_{e}$), and number density of solution species ($n_{l}$, $l=c,a,s$ for cation, anion, and solvent).

The expression for $\Omega$ reads

| $\Omega=\int gd^{3}r=\int\left( f_{Q}+f_{C}+f_{\mathrm{int}}-n_{e}\tilde{\mu}_{e}-\sum_{l=1}^{N_{C}} n_{l}\tilde{\mu}_{l} \right)d^{3}r,$ | (S1) |
| --- | --- |

where $f_{Q}$, $f_{C}$ and $f_{\mathrm{int}}$ represent the three contributions to the Helmholtz free energy, namely the quantum part for the metal electrons, the classical part for charged species in electrolyte, and the short-range interactions between metal electrons and classical charged species, respectively. Three modifications to $f_{C}$ has been introduced in Ref. [5] compared with Ref. [4] have been introduced, namely expressions for solvent concentration, concentration-dependent dipole moment, and specific metal–solvent interactions. Expressions for $f_{Q}$, $f_{C}$ and $f_{\mathrm{int}}$ will be presented in the following paragraphs. As only valence electrons are explicitly considered in $n_{e}$ (10 electrons per Au and Ag atom), the number density of metal atomic-core charges $n_{\mathrm{cc}}$ corresponds to 10 positive charges per atom, homogeneously distributed according to a jellium model. Furthermore, a very simple homogeneous pseudopotential within the jellium is utilized to describe the interactions between metal atomic-core charges and valence electrons. $\tilde{\mu}_{l}$ and $\tilde{\mu}_{e}$ are the electrochemical potentials of solution species and metal electrons. The electrode potential is linked to $\tilde{\mu}_{e}$ through $U \left( vs. SHE \right)=-(\tilde{\mu}_{e}-\tilde{\mu}_{e,SHE})/e$, where $\tilde{\mu}_{e,SHE}$ is the absolute potential of the SHE, assumed here as $\tilde{\mu}_{e,SHE}=-4.44$ eV [6].

$f_{Q}$ is given by the theory of inhomogeneous electron gas, [7] expressed as follows, [8,9]

| $f_{Q} =t_{\mathrm{ni}}\left[ n_{e},\nabla n_{e},\ldots\right]+u_{X}\left[ n_{e},\nabla n_{e},\ldots\right]+u_{C}\left[ n_{e},\nabla n_{e},\ldots\right]+p_{\mathrm{cc}}[n_{e}],$ | (S2) |
| --- | --- |

where $t_{\mathrm{ni}}\left[ n_{e},\nabla n_{e},\ldots\right]$ is the kinetic energy of non-interacting (subscript “ni”) electrons, $u_{X}\left[ n_{e},\nabla n_{e},\ldots\right]$ and $u_{C}\left[ n_{e},\nabla n_{e},\ldots\right]$ are the exchange and correlation energies, and $p_{\mathrm{cc}}[n_{e}]$ is the pseudopotential energy.

$t_{\mathrm{ni}}$ is given by the Thomas-Fermi-von Weizsäcker theory, [7,10,11]

| $t_{\mathrm{ni}}=e_{\mathrm{au}}a_{0}^{-3}t_{\mathrm{TF}}(1+\theta_{T}s^{2}),$ | (S3) |
| --- | --- |

where $t_{\mathrm{TF}}=\frac{3}{10}\left( 3\pi^{2} \right)^{\frac{2}{3}}\left( n_{e}a_{0}^{3} \right)^{\frac{5}{3}},$ $(1+\theta_{T}s^{2})$ is the correction for gradient terms, $s=\left| \nabla n_{e} \right|/(2\left( 3\pi^{2} \right)^{\frac{1}{3}}\left( n_{e} \right)^{\frac{4}{3}})$ is the reduced gradient term, and $\theta_{T}$ is a parameter tuning the contribution of the gradient term. The term $e_{\mathrm{au}}a_{0}^{-3}$ transforms the expression from atomic units to SI units, with $e_{\mathrm{au}}=e_{0}^{2}/(4\pi a_{0}\epsilon_{0})$ the atomic energy, $a_{0}$ the Bohr radius, $\epsilon_{0}$ the vacuum permittivity, and $e_{0}$ the unit of electrical charge. Here, $\theta_{T}=0.75$.

$u_{X}$ is written as,

| $u_{X}=e_{\mathrm{au}}a_{0}^{-3}u_{X}^{0}\left( 1+\theta_{X}s^{2} \right),$ | (S4) |
| --- | --- |

with $u_{X}^{0}=-\frac{3}{4}\left( \frac{3}{\pi} \right)^{\frac{1}{3}}\left( n_{e}a_{0}^{3} \right)^{\frac{4}{3}}$ the volumetric exchange energy of a uniform electron gas. $\theta_{X}$ is the parameter tuning the contribution of the gradient term in the exchange energy.

$u_{C}$ is given by

| $u_{C}=e_{\mathrm{au}}a_{0}^{-3}(u_{C}^{0}+\theta_{C}n_{e}a_{0}^{3}t^{2}),$ | (S5) |  |
| --- | --- | --- |

where $t=a_{0}^{4}\left| \nabla n_{e} \right|/\left( 4\left( \frac{3}{\pi} \right)^{\frac{1}{6}}\left( n_{e}a_{0}^{3} \right)^{\frac{7}{6}} \right)$ is another reduced density gradient in terms of dimensional $n_{e}$ and coordinates, and $u_{C}^{0}$ is the volumetric correlation energy of a uniform electron gas, for which the interpolation relation of Perdew et al. is used, [9]

| $u_{C}^{0}=-2\alpha_{1}n_{e}a_{0}^{3}\left( 1+\alpha_{2}r_{s} \right)\ln\left( 1+\frac{1}{\xi} \right),$  $r_{s}=\left( \frac{3}{4\pi n_{e}a_{0}^{3}} \right)^{\frac{1}{3}},$  $\xi=2\alpha_{1}\left( \alpha_{3}r_{s}^{\frac{1}{2}}+\alpha_{4}r_{s}+\alpha_{5}r_{s}^{\frac{3}{2}}+\alpha_{6}r_{s}^{2} \right),$ | (S6) |
| --- | --- |

with $\theta_{C}=0.046,\alpha_{1}=0.0310907, \alpha_{2}=0.21370,\alpha_{3}=7.5957,\alpha_{4}=3.5876,\alpha_{5}=1.6382,\alpha_{6}=0.49294$.

The volumetric pseudopotential energy is given by

| $p_{\mathrm{cc}}=e_{\mathrm{au}}v_{\mathrm{cc}}n_{e},$ | (S7) |  |
| --- | --- | --- |

where $v_{\mathrm{cc}}$ is the pseudopotential in atomic units, uniformly distributed within the jellium. In the subsequent section, we will show that $v_{\mathrm{cc}}$ can be calibrated to reproduce the experimental work function.

$f_{\mathrm{int}}$ describes specific interactions between solution particles and metal electron, which is be formulated as

| $f_{\mathrm{int}}=\sum_{l=1}^{N_{C}} n_{l}w_{l}\left( r \right),$  $w_{l}\left( r \right)=D_{l}\exp\left( -2\beta_{l}\left( d(r)-d_{l} \right) \right),$ | (S8) |
| --- | --- |

where $w_{l}\left( r \right)$ has the repulsive part of the Morse potential, with $D_{l}$ being the well depth, $\beta_{l}$ a coefficient for the well width, $d$ the distance between molecules and the metal surface, and $d_{l}$ the equilibrium distance between the molecule and the metal surface. Here, $D_{l}=0.0417 \mathrm{eV}, \beta_{l}=1, d_{l}=8.9792a_{0}$ (l=c,a) for cation and anion, and $D_{s}=0.25 \mathrm{eV}, \beta_{s}=1, d_{s}=1.8904a_{0}$ for solvent.

The classical contributions of free energy, $f_{C}$, is formulated as,

| $f_{C}=-\frac{1}{2}\epsilon_{\mathrm{opt}}\left( \nabla\phi\right)^{2}+\left( n_{c}-n_{a} \right)e\phi-n_{s}\beta^{-1}\ln\left( \frac{\sinh\left( \beta\left\vert\vec{p} \right\vert\left\vert\vec{E}_{\mathrm{tot}} \right\vert\right)}{\beta\left\vert\vec{p} \right\vert\left\vert\vec{E}_{\mathrm{tot}} \right\vert} \right)+\sum_{l=1}^{N_{C}} \beta^{-1}\left( n_{l}\ln\left( n_{l}\Lambda_{l}^{3} \right)-n_{l} \right)+\Phi_{\mathrm{ex}}\left( \left\{ n_{l} \right\} \right)+\left( n_{\mathrm{cc}}-n_{e} \right)e_{0}\phi.$ | (S9) |
| --- | --- |

Here, the first term on the right-hand side is the self-energy of the electric field, with $\epsilon_{\mathrm{opt}}$ the optical permittivity, $\phi$ the electric potential. The second and third terms represent the potential energies of charged particles in solution, with $n_{l} (l=c, a, s)$ the number density of cation, anion and solvent, and $\beta=\left( k_{B}T \right)^{-1}$ the inverse thermal energy. $\vec{p}$ is solvent dipole moment and $\vec{E}_{\mathrm{tot}}$ is the *effective total field*. The fourth term represents the Gibbs free energy of an ideal-gas reference system, with $\Lambda_{l}$ being the thermal wavelength of particle $l$. The fifth term $\Phi_{\mathrm{ex}}\left( \left\{ n_{l} \right\} \right)$ accounts for excess Gibbs free energy of the studied system compared with the ideal gas system, which accounts for finite-size effects, etc. For calculation of $\Phi_{\mathrm{ex}}$, see the Huang model in Ref. [12]. The sixth term represents the Hartree energy of electrons and cationic cores of the electrode.

Importantly, we have introduced three modifications compared to Ref. [4] that concern the expressions for solvent concentration ($c_{s}=n_{s}/N_{A}$), solvent dipole moment ($\vec{p}$) and the *effective total field* ($\vec{E}_{\mathrm{tot}}$) imposed on solvent dipoles. These are presented in detail in the subsequent paragraphs.

#### Modification of the expression for solvent concentration $\boldsymbol{c}_{\mathbf{s}}$

Since the first layer of solvents locates several Angstroms away from the electrode surface, we assume the first layer solvent to be centered at $L_{s}$. In Ref. [4], the local solvent concentration, $c_{s}=n_{s}/N_{A}$, can change in response to the electric field. In principle, $c_{s}$ can be determined self-consistently within the DPFT formalism. However, such a treatment sometimes leads to unphysically high local solvent densities and, consequently, large local dielectric constants (e.g., $\varepsilon_{s}>100$) in the vicinity of the electrode surface. To avoid this artifact, we adopted the same treatment as in our recent publication [5], in which the solvent concentration is fixed at its bulk value ($c_{s}^{b}=$55.6 mol/L) throughout the liquid region. Only within a narrow interfacial layer ($L_{s}$) does $c_{s}$ smoothly transition from zero to $c_{s}^{b}$, ensuring numerical stability and physical consistency without affecting the essential electrostatic features of the double layer. The width of the transition region is described by $l_{s}$,

| $c_{s}=\frac{c_{s}^{b}}{2}*\left( 1-\mathrm{erf} \left( -\frac{z-L_{s}}{l_{s}} \right) \right).$ | (S10) |
| --- | --- |

The metal–water distance $L_{s}$ is the width of the gap between the outmost atomic plane of a specific metal and the plane of the first solvent layer. Within this gap, the relative permittivity is low, *viz.*, $\epsilon_{r}=1$. The parameter, $L_{s}$, is closely related to the potential of zero charge (PZC) and $C_{d}$. Consequently, it can be calibrated to reproduce experimental data of PZC and $C_{d}$.

#### Dependence of effective dipole moment on ion concentration

In this study, we assume a negative correlation between the effective dipole moment of water ($p_{s}$) with local ion concentration ($c_{c}=n_{c}/N_{A}$, $c_{a}=n_{a}/N_{A}$), formulated as,

| $p_{s}=p_{w}\left( 1-\lambda(c_{c}+c_{a})/2 \right).$ | (S11) |
| --- | --- |

The relationship between the dielectric constant ($\epsilon_{s}$) and dipole moment takes the form of $\epsilon_{s}=\epsilon_{opt,s}+\frac{c_{s}N_{A}{p_{s}}^{2}}{3\epsilon_{0}k_{B}T}$. The negative correlation between $p_{s}$ and ion concentration can capture the dielectric decrement with high ion concentration [13]. Physically, this approach takes into account that a higher local ion concentration enhances ion–solvent interactions and thus leads to an effective reduction of the dielectric response of the solvent. Since the dielectric decrement effect affects the distance between the two local maxima in $C_{d}$ curves, $\lambda$ is also calibrated to reproduce the experimentally measured $C_{d}$. The calibration process is provided in detail in a subsequent section.

#### Incorporation of specific metal–solvent interactions into DPFT

Our model is able to describe the electrostatic interactions between the spillover electrons and the first-layer water molecules, leading to (i) a decrease of interfacial potential drop due to increased dielectric permittivity, and (ii) reorientation of water dipoles [14,15]. Given the orbital-free feature of DPFT, the electron exchange resulted from hybridization of metal surfaces and water molecules is not explicitly modelled [14,16,17]. In response, we take a heuristic approach to address this issue by introducing a new term, $H_{\mathrm{spe}c}$, which, together with the electrostatic term, $H_{\mathrm{elec}}$, jointly constitutes the solvent Hamiltonian,

| $H_{s}=H_{\mathrm{elec}}+H_{\mathrm{spe}c}.$ | (S12) |
| --- | --- |

$H_{\mathrm{spec}}$ represents an effective dipole–field interaction,

| $H_{\mathrm{spec}}=-\vec{p}\cdot\vec{A}$, | (S13) |
| --- | --- |

where the auxiliary field $\vec{A}$ is specific for a given electrode composition and surface crystal orientation. $\vec{A}$ is characterized by the magnitude, $A$, and direction, $\vec{n}$, *via* $\vec{A}=A\vec{n}$ with $\vec{n}=\left( n_{r}, n_{z} \right)$ being the unit vector pointing outwards from the solid surface and $A$ as the following function of distance to the surface, $d$,

| $A=\frac{A^{0}}{2}\left( 1+\mathrm{erf} \left( -\frac{d-L_{A}}{l_{A}} \right) \right),$ | (S14) |
| --- | --- |

where $L_{A}$ and $l_{A}$ are parameters representing the position and the width of the decay region. $A^{0}$, $L_{A}$ and $l_{A}$ are calibrated to reproduce the experimentally measured PZC.

For the derivation of the free energy functional of solvent molecules, we define the *total field* on a solvent molecule as $E_{\mathrm{tot}}=\vec{E}+\vec{A}$.

| $\vec{E}_{\mathrm{tot}}=\vec{E}+\vec{A}=\left( -\nabla_{r}\phi+An_{r},-\nabla_{z}\phi+An_{z} \right)$. | (S15) |
| --- | --- |

$H_{s}$ is rewritten as,

| $H_{s}=-\vec{p}\cdot\vec{E}_{\mathrm{tot}}=\left\vert p \right\vert\left\vert\vec{E}_{\mathrm{tot}} \right\vert\cos\omega,$ | (S16) |
| --- | --- |

where $\omega$ is the angle between $\vec{p}$ and $-\vec{E}_{\mathrm{tot}}$.

From now on, the derivation follows the same steps as in Ref. [4]. The partition function for the ensemble of solvent molecules reads,

| $Q_{s}=\left( \frac{\int_{0}^{\pi} \exp\left( -\frac{H_{s}}{k_{B}T} \right)\sin\omega d\omega}{\int_{0}^{\pi} \sin\omega d\omega} \right)^{n_{s}}=\left( \frac{\sinh\left( \beta\left\vert\vec{p} \right\vert\left\vert\vec{E}_{\mathrm{tot}} \right\vert\right)}{\beta\left\vert\vec{p} \right\vert\left\vert\vec{E}_{\mathrm{tot}} \right\vert} \right)^{n_{s}}.$ | (S17) |
| --- | --- |

The free energy functional of solvent molecules reads,

| $f_{s}=k_{B}T\ln Q_{s}=n_{s}\beta^{-1}\ln\left( \frac{\sinh\left( \beta\left\vert\vec{p} \right\vert\left\vert\vec{E}_{\mathrm{tot}} \right\vert\right)}{\beta\left\vert\vec{p} \right\vert\left\vert\vec{E}_{\mathrm{tot}} \right\vert} \right).$ | (S18) |
| --- | --- |

#### Derivation of controlling equations by minimization of the grand potential

In this study, the controlling equations for $n_{e}$ and $n_{l} (l=c,a,s)$ remain the same as in Ref. [4], while the controlling equation for $\phi$ takes a different form due to the replacement of $\vec{E}_{\mathrm{elec}}$ by $\vec{E}_{\mathrm{tot}}$ in $H_{s}$.

Employing the Euler-Lagrange equation with $\phi$ as the variational variable, we arrive the modified Poisson–Boltzmann equation,

| $-\nabla\cdot\left( \epsilon_{\mathrm{eff}}\nabla\phi\right)=e_{0}\left( n_{\mathrm{cc}}-n_{e} \right)+e_{0}\left( n_{c}-n_{a} \right)-\nabla\cdot\left[ n_{s}\left\vert\vec{p} \right\vert\frac{\mathcal{L}\left( \beta\left\vert\vec{p} \right\vert\left\vert\vec{E}_{\mathrm{tot}} \right\vert\right)}{\left\vert\vec{E}_{\mathrm{tot}} \right\vert}\vec{A} \right],$ | (S19) |
| --- | --- |

with $\mathcal{L}\left( x \right)=\coth\left( x \right)-\frac{1}{x}$ being the Langevin function.

This equation deviates from the traditional modified Poisson–Boltzmann equation in two ways: (i) an additional term for the dipolar solvent $-\nabla\left[ n_{s}\left| \vec{p} \right|\frac{\mathcal{L}\left( \beta\left| \vec{p} \right|\left| \vec{E}_{\mathrm{tot}} \right| \right)}{\left| \vec{E}_{\mathrm{tot}} \right|}\vec{A} \right]$, and (ii) the expression of $\epsilon_{\mathrm{eff}}$. The new equation for $\epsilon_{\mathrm{eff}}$ is

| $\epsilon_{\mathrm{eff}}=\epsilon_{\mathrm{opt}}+n_{s}\left\vert\vec{p} \right\vert\frac{\mathcal{L}\left( \beta\left\vert\vec{p} \right\vert\left\vert\vec{E}_{\mathrm{tot}} \right\vert\right)}{\left\vert\vec{E}_{\mathrm{tot}} \right\vert}.$ | (S20) |
| --- | --- |

where $\epsilon_{\mathrm{opt}}$ is written as,

| $\epsilon_{\mathrm{opt}}=\epsilon_{0}+\left( \epsilon_{opt,s}-\epsilon_{0} \right)n_{s}/n_{\mathrm{site}}.$ | (S21) |
| --- | --- |

with $\epsilon_{opt,s}=3.25\epsilon_{0}$ the optical dielectric constant of solvent.

The number density distribution of particles in the electrolyte solution is solved by applying $\frac{\partial g}{\partial n_{l}}-\nabla\left( \frac{\partial g}{\partial\left( \nabla n_{l} \right)} \right)=0$ and doing a few rearrangements [4,12]. The final expressions for $n_{c}$ and $n_{a}$ are

| $\frac{n_{c}}{n_{\mathrm{site}}}=\frac{n_{c}^{b}\exp\left[ -\beta\left( w_{c}+e\phi\right) \right]}{\gamma_{c}n_{c}^{b}\exp\left[ -\beta\left( w_{c}+e\phi\right) \right]+\gamma_{a}n_{a}^{b}\exp\left[ -\beta\left( w_{a}-e\phi\right) \right]+n_{\mathrm{site}}-\gamma_{c}n_{c}^{b}-\gamma_{a}n_{a}^{b}},$ | (S23) |
| --- | --- |
| $\frac{n_{a}}{n_{\mathrm{site}}}=\frac{n_{a}^{b}\exp\left[ -\beta\left( w_{a}-e_{0}\phi\right) \right]}{\gamma_{c}n_{c}^{b}\exp\left[ -\beta\left( w_{c}+e\phi\right) \right]+\gamma_{a}n_{a}^{b}\exp\left[ -\beta\left( w_{a}-e\phi\right) \right]+n_{\mathrm{site}}-\gamma_{c}n_{c}^{b}-\gamma_{a}n_{a}^{b}}.$ | (S24) |

with $\gamma_{c}$ and $\gamma_{a}$ the relative size of cation and anion with respect to a unit lattice site. $n_{c}^{b}$ and $n_{a}^{b}$ are the number density of cation and anion in bulk solution, respectively.

#### Boundary conditions

At the upper boundary in the bulk solution, we set $n_{e}$ and $\phi$ to zero. The lower boundary of the cell, located in the bulk of the support, as well as the boundary at $r=R_{\mathrm{cell}}$, demands a zero-gradient condition in normal directions for $n_{e}$ and $\phi$.

## Three-step parameter calibration

The calibration of model parameters for any specific electrode-electrolyte interface involves three consecutive steps to get (i) electronic, (ii) dielectric and (iii) ionic properties. In the first step, we calibrate parameters for electronic properties in the electrode-vacuum system to reproduce experimentally observed work function, $\Phi$. Then, we introduce a dielectric medium into the system and calibrate solvent parameters to reproduce the PZC. Last but not least, we incorporate electrolyte and calibrate ionic parameters to reproduce the $C_{d}$ curve. The calibration process is summarized in Table S1.

Table S1: Three-step calibration process

| **Step** | **Interface** | **Properties** | **Calibrated parameters** |
| --- | --- | --- | --- |
| **1** | solid–vacuum | work function | $v_{\mathrm{cc}}^{0}$ |
| **2** | solid–solvent | potential of zero charge | $L_{s},l_{s},A^{0},L_{A},l_{A}$ |
| **3** | solid–electrolyte | differential capacitance | $r_{a},r_{c},\lambda$ |

Table S2: Experimental literature references for Φ, PZC, and $C_{d}$ for calibration of parameters.

| **Electrode** | $\boldsymbol{\Phi}$ **[eV]** | **PZC [V_SHE_]** | $\boldsymbol{C}_{\mathbf{d}}$ |
| --- | --- | --- | --- |
| **pcAu** | 5.20  [18,19] | 0.19  [20,21] | [19,20] |
| **Ag(111)** | 4.60  [16,22,23] | -0.44  [24] | [24] |

#### Calibration of electronic parameters: reproduction of the work function

The work function, $\Phi=e\chi-\mu_{e}$, is constituted of the chemical potential of electrons, $\mu_{e}$, and the surface potential, $\chi$. $\mu_{e}$ is determined by the volumetric number density of metal core charges, $n_{\mathrm{cc}}$, and the pseudopotential caused by them, $v_{\mathrm{cc}}$. $\chi$ is influenced by $n_{\mathrm{cc}}$. Since $n_{\mathrm{cc}}$ is fixed for a specific material, we use $v_{\mathrm{cc}}$ as a fitting parameter to reproduce experimentally measured values of $\Phi$, as shown in Table S2.

As in Ref. [4], $n_{\mathrm{cc}}$ is calculated by $n_{\mathrm{cc}}=N_{e}n_{\mathrm{core}}=N_{e}\rho N_{A}/M$ with volumetric number density of metal atomic cores, $n_{\mathrm{core}}$, number of valence electrons per atom, $N_{e}$, mass density, $\rho$, and the Molar mass, $M$. Parameter values are shown in Table S3. Atom densities for both metal ($n_{\mathrm{core}}$) are around 60 $atom/\mathrm{nm}^{3}$.

Table S3: Calibrated electronic parameter values for pcAu and Ag(111) electrodes.

| **Slab** | $\boldsymbol{M [}\mathbf{g/mol}\boldsymbol{]}$ | $\boldsymbol{\rho[}\mathbf{g/}\mathbf{cm}^{\mathbf{3}}\boldsymbol{]}$ | $\boldsymbol{n}_{\mathbf{core}}\boldsymbol{[}\mathbf{c}\mathbf{m}^{\mathbf{-3}}\boldsymbol{]}$ | $\boldsymbol{N}_{\mathbf{e}}$ | $\boldsymbol{n}_{\mathbf{cc}}\boldsymbol{[}\mathbf{c}\mathbf{m}^{\mathbf{-3}}\boldsymbol{]}$ | $\boldsymbol{v}_{\mathbf{cc}}^{\boldsymbol{0}}\boldsymbol{[}\mathbf{eV}\boldsymbol{]}$ | $\boldsymbol{l}_{\boldsymbol{v}_{\mathbf{cc}}}\boldsymbol{[Å]}$ |
| --- | --- | --- | --- | --- | --- | --- | --- |
| **pcAu** | 197 | 19.3 | 5.8528$\times{10}^{22}$ | 11 | 6.4875$\times{10}^{23}$ | -1.5640 | $0.529$ |
| **Ag(111)** | 108 | 10.5 | 5.8978$\times{10}^{22}$ | 11 | 6.4381$\times{10}^{23}$ | 12.1312 | $0.529$ |

The decay of $v_{\mathrm{cc}}$ from the constant value in the metal bulk, $v_{\mathrm{cc}}^{0}$, to zero outside the metal surface is represented by the error function,

| $v_{\mathrm{cc}}=\frac{v_{\mathrm{cc}}^{0}}{2}\left( 1-\mathrm{erf} \left( -\frac{z}{l_{v_{\mathrm{cc}}}} \right) \right),$ | (S25) |
| --- | --- |

with the characteristic decay length $l_{v_{\mathrm{cc}}}$ assumed to be one Bohr radius. The calibration of $\Phi$ for Ag(111) and pcAu is completed by adjusting $v_{\mathrm{cc}}^{0}$, the values of which can be found in Table S3.

#### Calibration of solvent parameters: reproduction of the PZC

The calibration of solvent parameters requires setting the electrode potential at the PZC values (summarized in Table S2), and then tuning the solvent parameters, namely the profiles of $c_{s}$ and $\vec{A}$, to ensure the metal is at charge-neutral state. The calibrated values of the solvent parameters are summarized in Table S4. The selection of $L_{s}$ determines the value of the capacitance minimum at the PZC, as explained in the next section.

Table S4: Calibrated solvent parameter values for pcAu and Ag(111) electrodes.

| Symbol | Definition | Value | |
| --- | --- | --- | --- |
|  |  | pcAu | Ag(111) |
| $\boldsymbol{L}_{\mathbf{s}}$ | position of the edge for solvent concentration, [$Å$] | 1.1295 | 0.8000 |
| $\boldsymbol{l}_{\mathbf{s}}$ | width of the edge for solvent concentration, [$Å$] | 0.1 | 0.1 |
| $\boldsymbol{A}$^0^ | strength of the chemical interaction, [$V/m$] | 0 | -22.185 |
| $\boldsymbol{L}_{\mathbf{A}}$ | position of the edge for $\vec{A}$, $[Å]$ | 3.9295 | 3.6000 |
| $\boldsymbol{l}_{\mathbf{A}}$ | width of the edge for $\vec{A}$, $[Å]$ | 0.1 | 0.1 |

#### Calibration of ionic parameters: reproduction of the capacitance curve

In the third step, we calibrate ionic parameters to reproduce $C_{d}$ curves. As shown in FIG. S1, $C_{d}$ curve has four features, namely (a) the value of the $C_{d}$ minimum, (b) the abscissa value of the $C_{d}$ minimum, (c) the height of right and left peaks, and (d) the distance between the peaks.

Feature (a) is determined by the width of the gap between the electrode surface and the first layer of solvent molecules. This is due to the gap region exhibiting the lowest dielectric constant and thus the lowest capacitance. Feature (b) corresponds to the PZC of the electrode–electrolyte interface [25]. Since both features (a) and (b) have been considered in calibration step 2, we are left with features (c) and (d). The relative height of the two peaks is determined by the cation and anion radii, whereas the distance between the two peaks is a joint result of dielectric decrement and volume exclusion.

The $C_{d}$ curves of pcAu in KClO_4_ solution and Ag(111) in KPF_6_ solution have been reproduced using fitting parameters in Table S5. Experimental data and fitting results are demonstrated in FIG. S1.

| 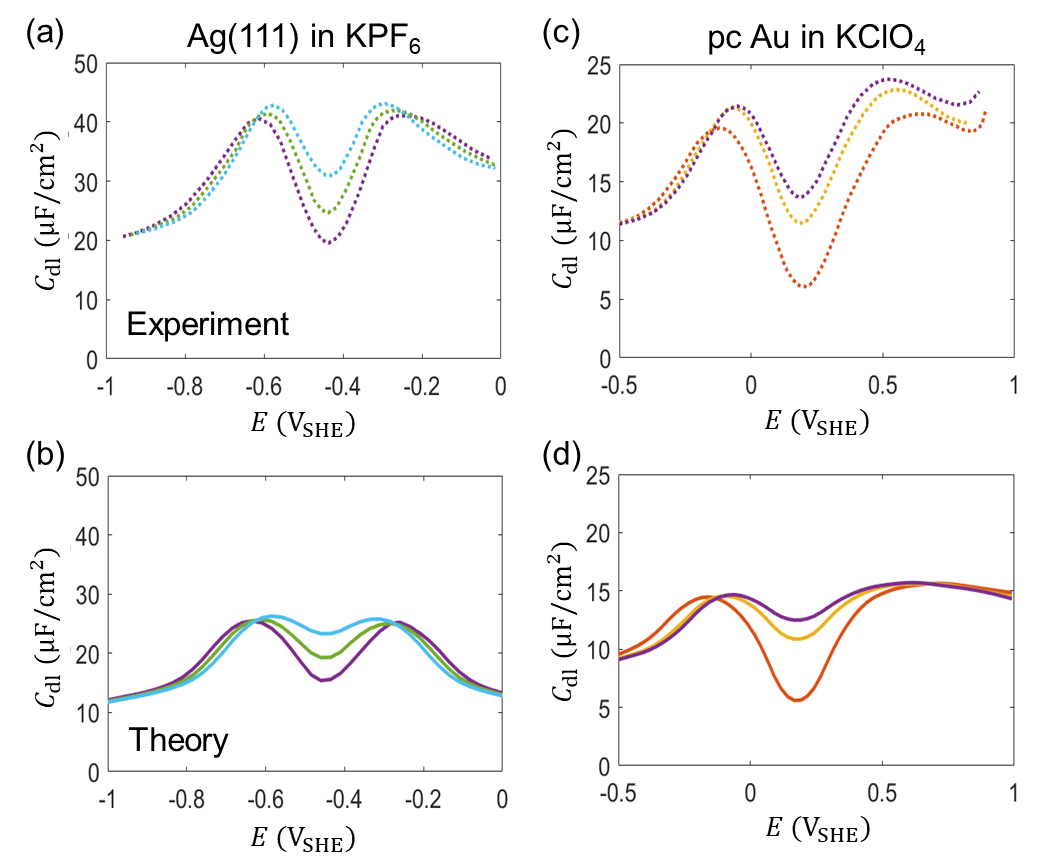 |
| --- |
| FIG. S1. $C_{d}$ curve for (a) Ag(111) in KPF_6_ solution of 40 mM (blue), 20 mM (green) and 10 mM (purple) and (c) pcAu in KClO_4_ solution of 20 mM (purple), 10 mM (yellow) and 1 mM (orange). (a) and (c) are experimental data [20,21,24] whereas (b) and (d) are fitting results. |

Table S5: Calibrated ionic parameter values for pcAu and Ag(111) electrodes.

| Symbol | Definition | Value | |
| --- | --- | --- | --- |
|  |  | pcAu | Ag(111) |
| $\boldsymbol{r}_{\mathbf{a}}$ | anion radius | 3.38 $Å$ ($\mathrm{ClO}_{4}^{-}$) | 5.5 $Å$ ($\mathrm{PF}_{6}^{-}$) |
| $\boldsymbol{r}_{\mathbf{c}}$ | cation radius | 6 $Å$ ($K^{+}$) | 5.5 $Å$ ($K^{+}$) |
| $\boldsymbol{\lambda}$ | dependence of the effective dipole moment on local ion concentration | $7.255\times{10}^{-4}$ $\mathrm{mol}^{-1}$ | $9.481\times{10}^{-4}$ $\mathrm{mol}^{-1}$ |

## Equilibrium distance between nanoparticle and support

We performed a parametric sweep of the distance between NP and support in vacuum, and calculated the grand potential of the system. The minimum locates at a distance of 0.1 $\mathrm{nm}$, as shown in FIG. S2. The binding energy per atom is roughly 4 eV.

| 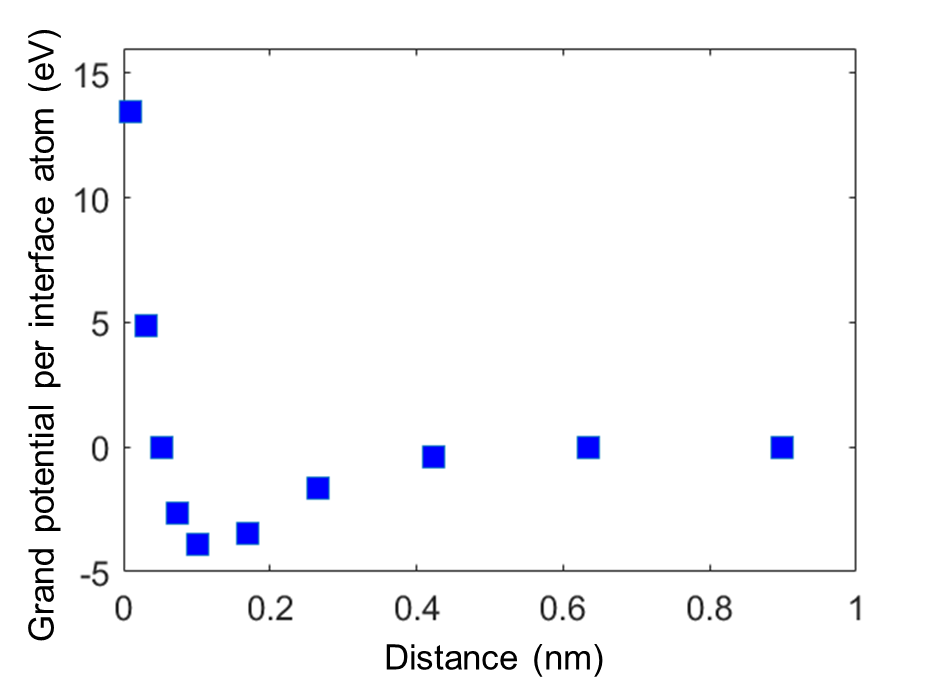 |
| --- |
| FIG. S2. Grand potential of the total system as a function of the gap distance between NP and support. The NP–support contact interface is a circle of 1 nm radius, corresponding to approximately 45 atoms for Ag(111) facet. The grand potential per interface atom is therefore normalized with respect to 45 atoms. |

## References

[1] J. Huang, Mixed quantum-classical treatment of electron transfer at electrocatalytic interfaces: Theoretical framework and conceptual analysis, J Chem. Phys. **153**, 164707 (2020).

[2] J. Huang, Hybrid density-potential functional theory of electric double layers, Electrochim. Acta **389**, 138720 (2021).

[3] J. Huang, S. Chen, and M. Eikerling, Grand-Canonical Model of Electrochemical Double Layers from a Hybrid Density–Potential Functional, J. Chem. Theory Comput. **17**, 2417 (2021).

[4] J. Huang, Density-Potential Functional Theory of Electrochemical Double Layers: Calibration on the Ag(111)-KPF6 System and Parametric Analysis, J. Chem. Theory Comput. **19**, 1003 (2023).

[5] Y. Zhang, T. Binninger, J. Huang, and M. H. Eikerling, Theory of Electro-Ionic Perturbations at Supported Electrocatalyst Nanoparticles, Phys. Rev. Lett. **134**, 066201 (2025).

[6] S. Trasatti, The absolute electrode potential: an explanatory note (Recommendations 1986), J. Electroanal. Chem. Interfacial Electrochem. **209**, 2 (1986).

[7] S. Lundqvist and N. H. March, *Theory of the Inhomogeneous Electron Gas* (Springer Science & Business Media, 2013).

[8] W. Kohn and L. J. Sham, Self-Consistent Equations Including Exchange and Correlation Effects, Phys. Rev. **140**, 4A (1965).

[9] J. P. Perdew and S. Kurth, *Density Functionals for Non-Relativistic Coulomb Systems in the New Century*, in *A Primer in Density Functional Theory*, edited by C. Fiolhais, F. Nogueira, and M. A. L. Marques (Springer Berlin Heidelberg, Berlin, Heidelberg, 2003), pp. 1–55.

[10] L. H. Thomas, The calculation of atomic fields, Math. Proc. Camb. Philos. Soc. **23**, 5 (1927).

[11] E. Fermi, Eine statistische Methode zur Bestimmung einiger Eigenschaften des Atoms und ihre Anwendung auf die Theorie des periodischen Systems der Elemente, Z. Physik **48**, 73 (1928).

[12] Y. Zhang and J. Huang, Treatment of Ion-Size Asymmetry in Lattice-Gas Models for Electrical Double Layer, J. Phys. Chem. C **122**, 28652 (2018).

[13] R. Buchner, G. T. Hefter, and P. M. May, Dielectric Relaxation of Aqueous NaCl Solutions, J. Phys. Chem. A **103**, 1 (1999).

[14] P. Li, J. Huang, Y. Hu, and S. Chen, Establishment of the Potential of Zero Charge of Metals in Aqueous Solutions: Different Faces of Water Revealed by Ab Initio Molecular Dynamics Simulations, J. Phys. Chem. C **125**, 3972 (2021).

[15] J. Le, Q. Fan, J. Li, and J. Cheng, Molecular origin of negative component of Helmholtz capacitance at electrified Pt(111)/water interface, Sci. Adv. **6**, eabb1219 (2020).

[16] J. Le, M. Iannuzzi, A. Cuesta, and J. Cheng, Determining Potentials of Zero Charge of Metal Electrodes versus the Standard Hydrogen Electrode from Density-Functional-Theory-Based Molecular Dynamics, Phys. Rev. Lett. **119**, 1 (2017).

[17] A. Groß and S. Sakong, Ab Initio Simulations of Water/Metal Interfaces, Chem. Rev. **122**, 10746 (2022).

[18] E. E. Huber Jr., The Effect of Mercury Contamination on the Work Function of Gold, Appl. Phys. Lett. **8**, 169 (2004).

[19] R. P. W. Lawson and G. Carter, The Desorption of Mercury and the Work Function of Polycrystalline Gold, Appl. Phys. Lett. **9**, 85 (2004).

[20] J. Clavilier and C. N. Van Huong, Etude de l’interface de l’or polycristallin au contact de solutions aqueuses de perchlorate de potassium et d’acide perchlorique, Journal of Electroanalytical Chemistry and Interfacial Electrochemistry **80**, 101 (1977).

[21] Z. Samec and K. Doblhofer, Mechanism of peroxodisulfate reduction at a polycrystalline gold electrode, J. Electroanal. Chem. **367**, 141 (1994).

[22] M Chelvayohan and C H B Mee, Work function measurements on (110), (100) and (111) surfaces of silver, J Phys. C: Solid State Phys. **15**, 2305 (1982).

[23] H. E. Farnsworth and R. P. Winch, Photoelectric Work Functions of (100) and (111) Faces of Silver Single Crystals and Their Contact Potential Difference, Phys. Rev. **58**, 812 (1940).

[24] G. Valette, Double layer on silver single crystal electrodes in contact with electrolytes having anions which are slightly specifically adsorbed: Part III. The (111) face, J. Electroanal. Chem. & Interfacial Electrochem. **269**, 191 (1989).

[25] A. A. Kornyshev, Double-Layer in Ionic Liquids:  Paradigm Change?, J. Phys. Chem. B **111**, 5545 (2007).
